# Structural basis of ferroportin inhibition by minihepcidin PR73

**DOI:** 10.1371/journal.pbio.3001936

**Published:** 2023-01-17

**Authors:** Azaan Saalim Wilbon, Jiemin Shen, Piotr Ruchala, Ming Zhou, Yaping Pan

**Affiliations:** 1 Verna and Marrs McLean Department of Biochemistry and Molecular Biology, Baylor College of Medicine, Houston, Texas, United States of America; 2 Department of Medicine, David Geffen School of Medicine, University of California Los Angeles, Los Angeles, California, United States of America; University of Zurich, SWITZERLAND

## Abstract

Ferroportin (Fpn) is the only known iron exporter in humans and is essential for maintaining iron homeostasis. Fpn activity is suppressed by hepcidin, an endogenous peptide hormone, which inhibits iron export and promotes endocytosis of Fpn. Hepcidin deficiency leads to hemochromatosis and iron-loading anemia. Previous studies have shown that small peptides that mimic the first few residues of hepcidin, i.e., minihepcidins, are more potent than hepcidin. However, the mechanism of enhanced inhibition by minihepcidins remains unclear. Here, we report the structure of human ferroportin in complex with a minihepcidin, PR73 that mimics the first 9 residues of hepcidin, at 2.7 Å overall resolution. The structure reveals novel interactions that were not present between Fpn and hepcidin. We validate PR73-Fpn interactions through binding and transport assays. These results provide insights into how minihepcidins increase inhibition potency and will guide future development of Fpn inhibitors.

## Abstract 2

Ferroportin is the only known iron exporter in humans, and its suppressed by hepcidin, an endogenous peptide hormone. The structure of human ferroportin bound to the hepcidin mimetic peptide PR73 reveals interactions not seen in the hepcidin-ferroportin complex and provides a rationale for the enhanced affinity of PR73.

## Introduction

Ferroportin (Fpn) is a Fe^2+^/2H^+^ antiporter that is highly expressed in enterocytes, hepatocytes, and macrophages to export Fe^2+^ derived from either dietary intake or digestion of senescent red blood cells [1–3]. Since Fpn is the only known iron exporter in mammals, its activity is essential for plasma iron homeostasis [4]. Ferroportin activity can be acutely suppressed by the peptide hormone hepcidin that binds to Fpn to inhibit iron transport. Binding of hepcidin also induces endocytosis and degradation of Fpn to further reduce iron transport [5,6]. Meanwhile, the expression of hepcidin is regulated via plasma iron levels, resulting in a closely monitored hepcidin-ferroportin axis [7,8]. Mutations in Fpn that impair transport activity can cause ferroportin disease, leading to symptoms of iron-deficiency anemia [9,10]. Elevated hepcidin levels can also lead to iron deficiency [11]. Hepcidin deficiency and hepcidin-resistant mutations in Fpn, on the other hand, lead to hereditary hemochromatosis and iron overload [10]. Thus, the hepcidin-ferroportin axis must be tightly regulated to maintain serum iron levels.

**Fig 1:**
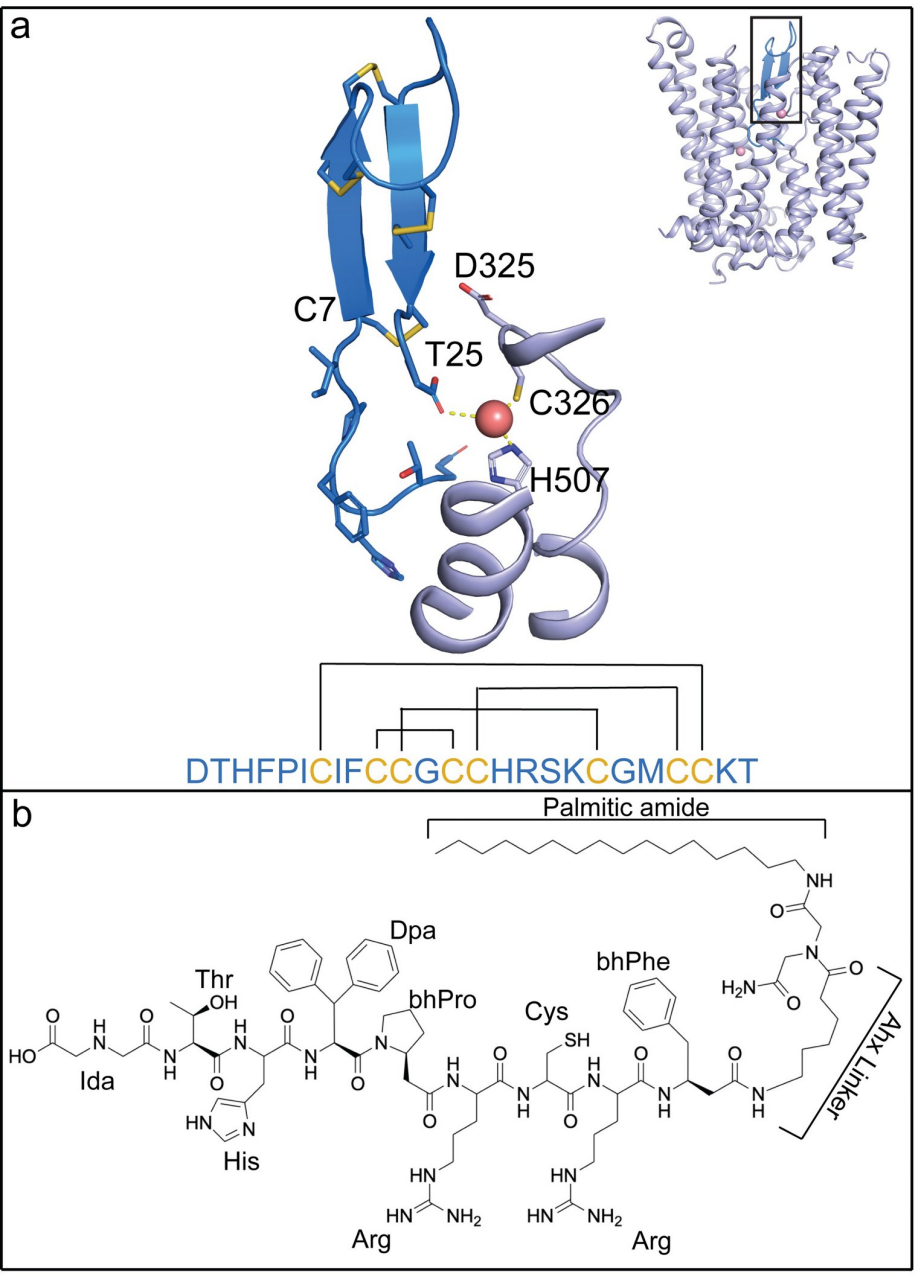
Structural comparison of hepcidin and PR73 molecules. (**a**) Structure of hepcidin in complex with HsFpn (PDB: 6WBV). Hepcidin (marine), HsFpn (light blue), and Co^2+^ (light pink) are shown in the overall (inset) and zoomed-in views. Side chains of the first seven residues and all cysteines in hepcidin are shown as sticks, and so is the carboxyl group of the last residue. The hepcidin sequence is shown as single-letter codes at the bottom, and the disulfide bridges are marked by black lines. (**b**) Chemical structure of PR73. Unnatural amino acid abbreviations are as follows: Ida, iminodiacetic acid; Dpa, diphenylalanine; bhPro, beta homo-proline; bhPhe, Beta homo-phenylalanine; Ahx, aminohexanoic linker.

Hepcidin is a peptide of 25 amino acids and is secreted by hepatocytes. Hepcidin binds to the extracellular side of Fpn [2,12,13]. There are 4 pairs of intramolecular disulfide bridges in hepcidin [14,15] (**Fig 1A**), and mutational studies showed that the first 7–9 residues have a large impact on its ability to inhibit Fpn [16]. The dissociation of the Fpn-hepcidin complex under reducing conditions led to the hypothesis that the disulfide bridge between Cys7 and Cys23 in hepcidin could be replaced with a disulfide bridge between Cys7 and Cys326 on Fpn [17], although this exchange was not observed in the structure of Fpn bound to hepcidin [12]. These studies led to the development of a new class of potent Fpn inhibitors, termed minihepcidins, that are based on the first 7–9 amino acids of hepcidin. Some of the minihepcidins have drastically improved potency to Fpn and have been further optimized and tested as hepcidin replacements to treat patients with dysregulation of hepcidin [18]. However, it remains unknown how the minihepcidins bind to Fpn with a higher affinity than hepcidin.

Structures of Fpn in complex with hepcidin revealed how the 2 interact [2,12]. Hepcidin binds between the N-terminal domain (NTD) and C-terminal domain (CTD) of Fpn, and the first 6 residues of hepcidin interact with Fpn. Hepcidin retains all 4 of its internal disulfide bonds, and its C-terminal carboxylate coordinates 1 metal ion at one of the binding sites, which is formed by Cys326 and His507 of Fpn [12]. Importantly, residue Cys326 is known to affect hepcidin binding and is associated with hemochromatosis [17,19]. These structures have presented insights into the binding mechanism of hepcidin to Fpn.

Minihepcidins are highly effective in inhibiting Fpn activity [20]. PR73 is a minihepcidin and a highly potent inhibitor of Fpn [21] whose predecessor showed effectiveness in preventing iron overload [18]. PR73 mimics the first 9 residues of hepcidin, including the cysteine in position 7 that was hypothesized to form a disulfide bridge with residue Cys326 of Fpn [17] (**Fig 1B**). The last 16 residues of hepcidin have been replaced with an aminohexanoic linker and iminodiacetic palmitic amide (Ida(NHPal)) in PR73, eliminating the C-terminal carboxylate essential for the binding of hepcidin. Thus, it is not clear how PR73 inhibits Fpn with increased potency.

Here, we present the structures of human (*Homo sapiens*) Fpn (HsFpn) bound to PR73 and Co^2+^ at 2.7 Å and 3.0 Å, respectively. We used a fragment of antigen-binding (Fab) to facilitate structure determination by cryo-electron microscopy (cryo-EM). The structures show that PR73 preserves most of the original interactions between hepcidin and Fpn. Although PR73 lacks the C-terminal carboxylate to coordinate the bound metal ion, it forms a disulfide bridge with HsFpn, and the palmitic amide of PR73 interacts with Gln194 on Fpn, which positions the acyl chain of PR73 in the hydrophobic core of the cell membrane surrounding Fpn.

## Results

### Structure of HsFpn in complex with 11F9 Fab

In a previous study, we reported the structure of the *Tarsius syrichta* ferroportin (TsFpn) in complex with an Fab from the mouse monoclonal antibody (11F9) [2]. The Fab is crucial for structure determination because it increases the size of the particle and serves as a fiduciary marker for particle alignment. Since TsFpn is 92% identical to human Fpn and 15 of the 16 residues that form the epitope are identical in the 2 Fpns (**S1A Fig**), we tested the binding of 11F9 Fab to HsFpn. The Fab binds to HsFpn with a dissociation constant (*K*_*D*_) of 10.6 ± 3.2 nM (**S1B Fig**). In addition, we showed that HsFpn and the Fab can form a stable complex after reconstitution into lipid nanodiscs (**S1C Fig**). The 11F9 Fab is known to inhibit TsFpn activity [2], and we found that the Fab also inhibits Co^2+^ and Fe^2+^ transport by HsFpn (**S1D–S1I Fig**).

**Fig 2:**
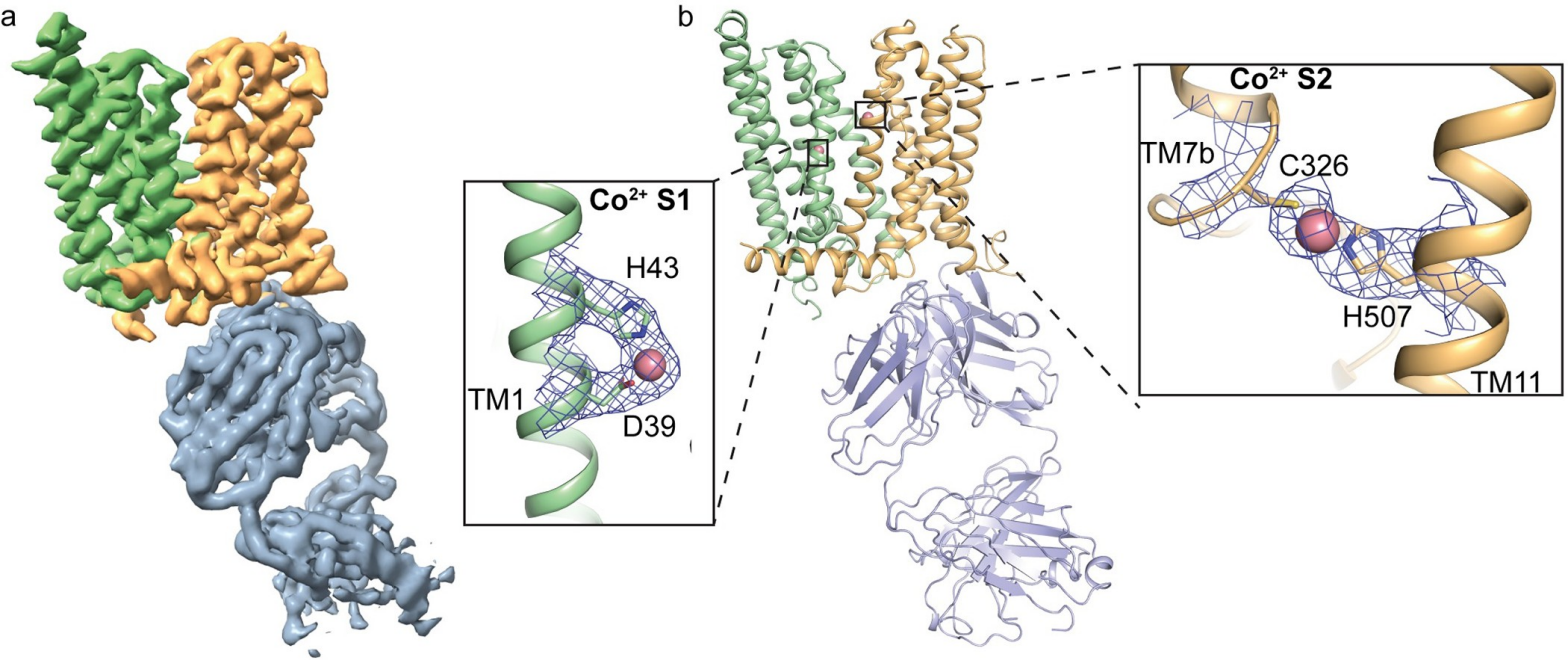
Structure of HsFpn in complex with 11F9 Fab. (**a**) Cryo-EM density map of HsFpn in complex with 11F9 Fab in the presence of Co^2+^. Densities for the NTD, CTD, and Fab are colored in pale green, light orange, and slate gray, respectively and contoured at 8.5σ. (**b**) Cartoon representation of HsFpn-11F9 complex. Ligand residues of the transition metal ion binding sites, S1 and S2, are highlighted and shown as sticks. Co^2+^ is rendered as a sphere (light pink). Densities for S1 and S2 are contoured at 4σ as blue mesh. CTD, C-terminal domain; cryo-EM, cryo-electron microscopy; Fab, fragment of antigen-binding; NTD, N-terminal domain.

Previous studies have shown both the capability and importance of Fpn transporting both cobalt and zinc [22,23]. In addition, structural and in vitro studies of Fpn have used Co^2+^ as a substitute for Fe^2+^ [2,12] due to the instability of Fe^2+^ in aerobic solution. Thus, we reconstituted the HsFpn-11F9 complex into lipid nanodiscs and determined the structure of Co^2+^-bound HsFpn at an overall resolution of 3.01 Å by cryo-EM (**Figs 2A** and **S2**). The density map allows the model building of all transmembrane (TM) helices and most of the side chains. Residues 15–236, 284–396, and 451–557 are resolved and included in the final structure (**S3 Fig**). Similar to previous structures of TsFpn and HsFpn, the current structure assumes an outward-facing conformation and aligns well with the previously reported human and monkey Fpn with a root-mean-squared distance (RMSD) of 0.57 Å and 0.58 Å, respectively (**S1A** and **S1J Fig**). Two Co^2+^ binding sites are resolved, with Site 1 (S1) composed of Asp39 and His43 and Site 2 (S2) of Cys326 and His507 (**Fig 2B**). In addition, the Fab interacts with both the CTD and NTD of HsFpn, similar to the previously determined structures of TsFpn-11F9.

### Cryo-EM structure of HsFpn bound to PR73

**Fig 3:**
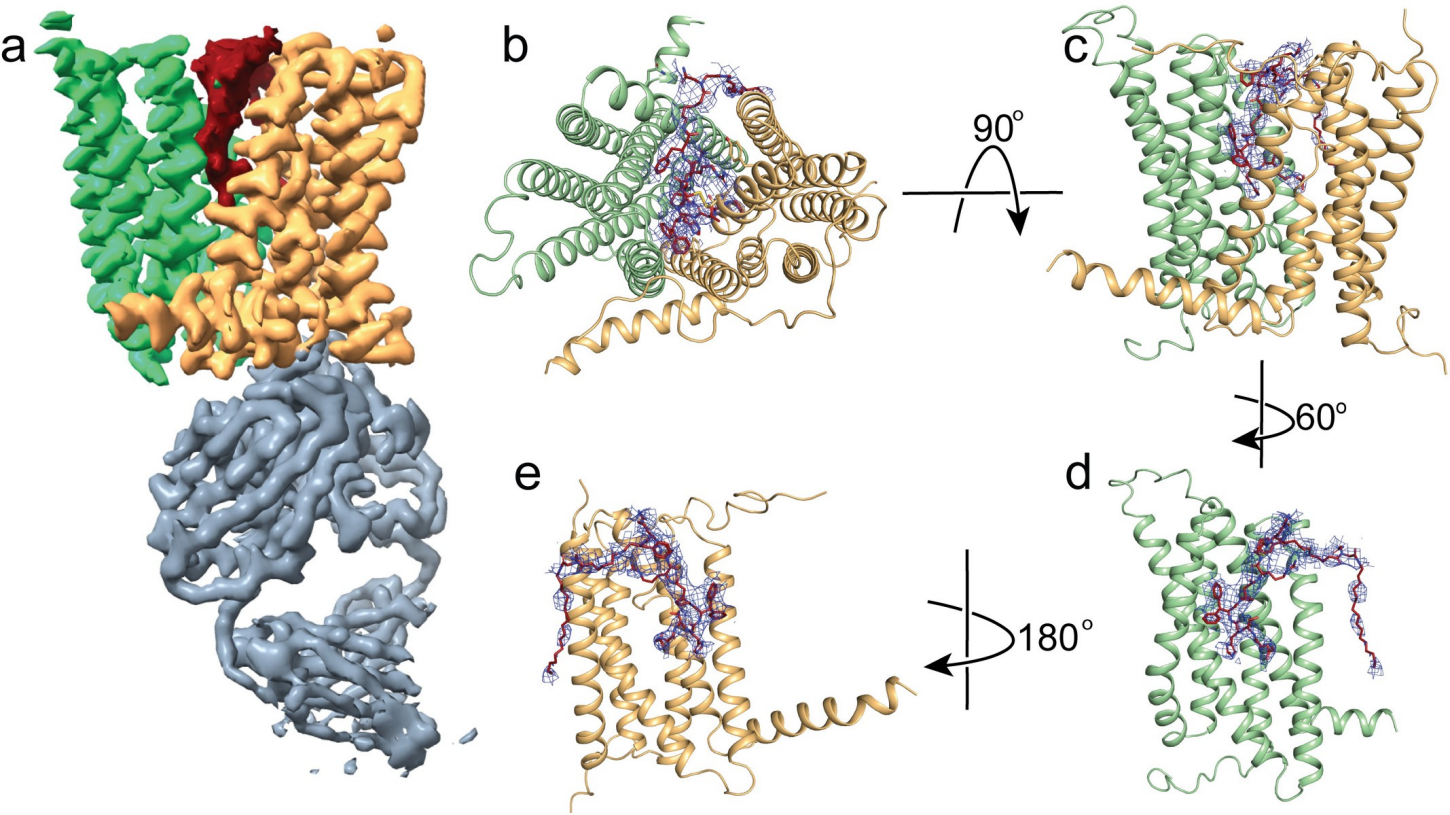
Structure of HsFpn bound to PR73. (**a**) Cryo-EM density map of HsFpn in complex with 11F9 Fab in the presence of PR73. Densities for the NTD, CTD, PR73, and Fab are colored in pale green, light orange, brick red, and slate gray, respectively. HsFpn and the Fab are contoured at 8.5σ while the PR73 density is contoured at 4σ. Extracellular (**b**) and side (**c**) views of the HsFpn-PR73 structure. HsFpn is shown as a cartoon, and PR73 is shown as sticks (brick red) with the density contoured at 4σ as blue mesh. Side views of HsFpn-PR73 with the CTD (**d**) or NTD (**e**) omitted. CTD, C-terminal domain; cryo-EM, cryo-electron microscopy; Fab, fragment of antigen-binding; NTD, N-terminal domain.

Next, we determined the structure of HsFpn-11F9 in nanodiscs in the presence of 1 mM of minihepcidin, PR73, to an overall resolution of 2.72 Å (**Figs 3A** and **S4**). The density map allows the modeling of residues 12–241, 283–416, and 449–558, which are included in the final structure (**S5 Fig**). Residues 1–11, 242–282, 417–448, and 559–571 are not resolved. There are 2 lipid densities located near 2 amphipathic helices (AHs), 1 in the NTD and another in the CTD, on the intracellular side (**S6 Fig**). A large non-protein density is present between the NTD and CTD towards the extracellular side and was absent in previous maps of HsFpn when PR73 was not included. The density accommodates PR73 in an extended conformation (**Fig 3B**–**3**E). The binding of PR73 seems to push the NTD and CTD away from each other, which resembles what was observed in the hepcidin-bound HsFpn and TsFpn structures.

**Fig 4:**
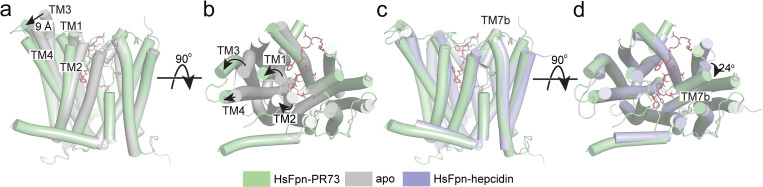
Structural changes induced by PR73. Structure of HsFpn-PR73 (pale green) aligned with apo-HsFpn (gray, PDB ID 6W4S) viewed from the side (**a**) and the extracellular side (**b**). Large structural changes are highlighted by arrows. Structures of HsFpn-PR73 aligned with HsFpn-Hepcidin (light blue, PDB ID 6WIK) viewed from the side (**c**) and extracellular side (**d**).

The overall conformation of the PR73-bound HsFpn structure is similar to those of the hepcidin-bound HsFpn and TsFpn, with a C_α_ RMSD of 0.82 Å and 1.10 Å, respectively (**Fig 4**). When the CTD of the current structure is aligned to that of HsFpn without an inhibitor, the extracellular sides of the N-domain, especially of TM1, TM2, TM3, and TM4, are pushed away from the CTD (**Fig 4A** and **4B**). The extracellular end of TM3 has the largest movement of approximately 9 Å, while those of TM1, TM2, and TM4 have movements of 4–5 Å. In the CTD, the largest change is around S2. Here, TM7b has a rotation of approximately 24° when compared to the hepcidin-bound structure, likely induced by the disulfide bridge between Cys326 and Cys7 of PR73. This rotation is unique to the PR73-bound structure because in the hepcidin-bound structure Cys326 maintains its interaction with the bound ion, which in turn interacts with the C-terminal carboxylate of hepcidin.

**Fig 5:**
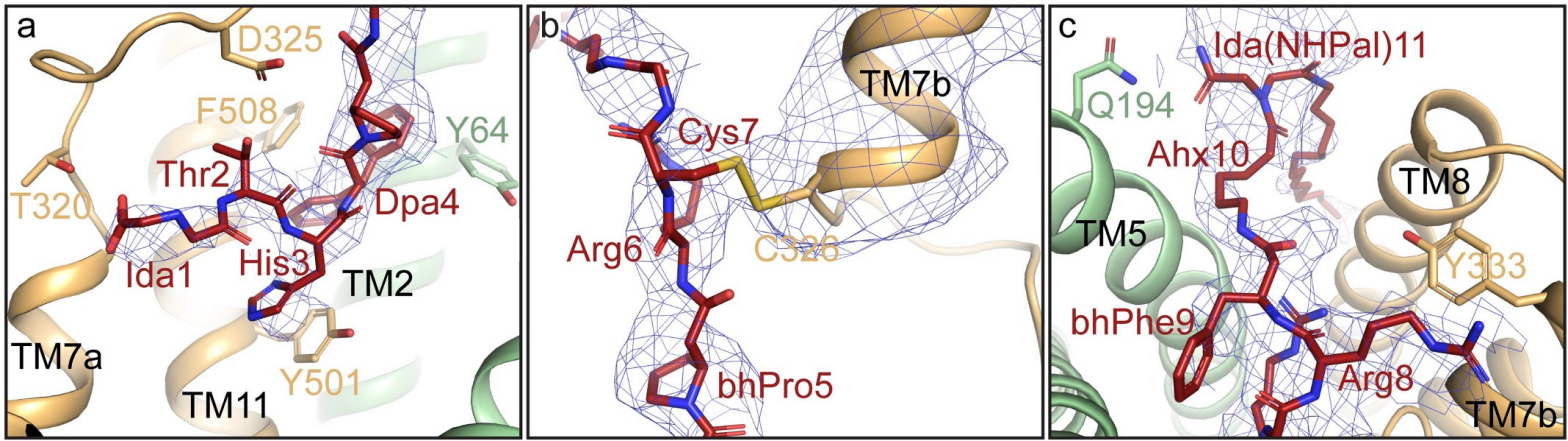
Interactions between PR73 and HsFpn. (**a**) Zoomed-in view of the first 4 residues of PR73 peptide. The density of PR73 is contoured at 4.5σ as blue mesh. Residues of HsFpn forming interactions with PR73 are shown as side-chain sticks. (**b**) Zoomed-in view of residues 5–7 of PR73 displayed in the same representation as in (**a**). The density of TM7b is contoured at 4.5σ as blue mesh. (**c**) Zoomed-in view of the last 4 residues of PR73 shown in the same representation as in (**a**).

Interactions between PR73 and HsFpn share common features with those of hepcidin and HsFpn, but there are several unique features as well. The first 6 residues of PR73 include native and modified versions of amino acids found in hepcidin. These residues interact with residues along TM7a and TM11 (**Figs 5A** and **S7**), including residues Tyr501 and Phe508 that extend towards the hydrophobic region of PR73 between Thr2 and Arg6. These interactions are similar in the structure of hepcidin-HsFpn. While the carboxylate at the C-terminus of hepcidin participates in the coordination of the bound metal ion to enhance hepcidin affinity, Cys7 of PR73 seems to form a disulfide bond with Cys326 of HsFpn. In the density map, the side chain of Cys326 (Fpn) and Cys7 (PR73) are in close proximity (**Fig 5B**). The connectivity of the side chain density is similar to the 3 native disulfide bridges, 1 in the CTD of HsFpn and 2 in 11F9 Fab (**S8 Fig**). The density map in conjunction with previous mutational studies that support a disulfide bridge formation between PR73 and Fpn [16,21] prompted us to build a disulfide bridge between Cys326 and Cys7. Additionally, Tyr333 of TM7b, which forms a hydrogen bond with Met21 of hepcidin, now has a potential cation-π interaction with Arg8 of PR73 (**Fig 5C**). Lastly, the aminohexanoic linker and palmitic amide tail of PR73 protrude out of the domain interface and insert into the membrane. This leads to an unexpected interaction where Gln194 on TM5 forms a hydrogen bond with the iminodiacetic palmitic amide (Ida(NHPal)) (**Fig 5C**). This interaction is also unique to PR73 as there is no evidence that Gln194 is involved in the hepcidin binding [12].

### Validation of PR73-Fpn interactions

**Fig 6:**
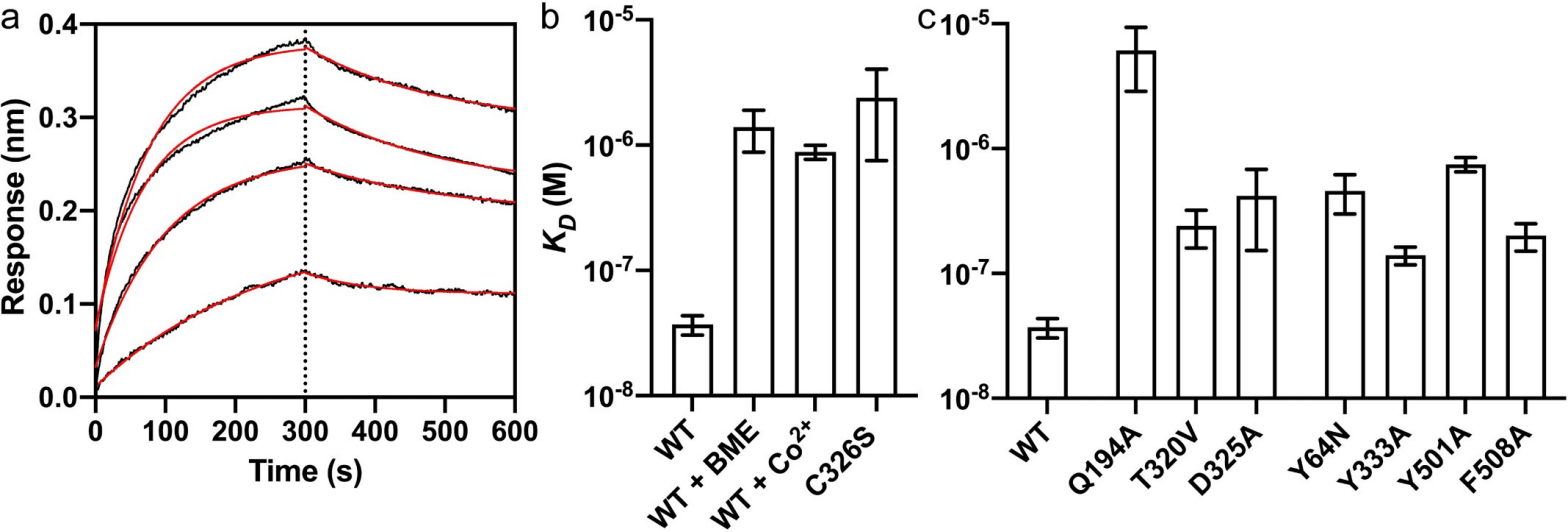
Binding of PR73 to Fpn. (**a**) Octet traces of WT Fpn in the presence of 1.25, 2.5, 5, and 10 μM PR73. The average *K*_*D*_ is approximately 37 nM. (**b**) Binding affinity of HsFpn to PR73 in different conditions, the presence of 5 mM BME, 5 mM Co^2+^, or the Cys326Ser mutant. (**c**) Binding affinity of HsFpn mutants to PR73. The height of the bars represents the mean of at least 3 measurements (*n* = 3) and the error bars the SEM. Source data for (**a**–**c**) can be found in **S1 Data**. BME, β-mercaptoethanol; SEM, standard error of the mean; WT, wild type.

PR73 is known to inhibit Fpn in the nanomolar range in cell-based assays [21]. We measured the binding affinity of PR73 to the purified HsFpn and found that the equilibrium *K*_*D*_ is approximately 37 nM (**Fig 6A** and **S2 Table**), which is consistent with previous results from cell-based assays [16,21]. Next, we examined the interactions between Cys7 of PR73 and Cys326 of HsFpn, which is unique to PR73. We first measured binding affinity in the presence of β-mercaptoethanol (BME), which would mask cysteine residues to prevent the formation of disulfide bridges (**Fig 6B** and **S2 Table**). The affinity is reduced by approximately 40-fold to *K*_*D*_ approximately 1.4 μM in the presence of BME, indicating that the cysteine residues contribute significantly to the binding affinity. We then measured the binding affinity of PR73 to the wild-type (WT) HsFpn in the presence of 5 mM CoCl_2_ (**Fig 6B**). Co^2+^ ion was shown to be required for the binding of hepcidin because the C-terminus carboxylate of hepcidin coordinates the ion with Cys326 [12]. In contrast, PR73 affinity is reduced in the presence of Co^2+^ (*K*_*D*_ approximately 880 nM), which we interpret as in the presence of Co^2+^, Cys326 would coordinate Co^2+^ and is less available to form a disulfide bridge with PR73. Finally, we measured the affinity of PR73 to the Cys326Ser mutant HsFpn and found that the affinity is significantly reduced (*K*_*D*_ approximately 2.4 μM). These results are consistent with formation of a disulfide bridge between PR73 and HsFpn; however, we cannot rule out the possibility that 2 cysteines are close enough to interact but do not form a covalent bond. In fact, we observed a measurable off-rate during the dissociation step of the binding assay (**Fig 6A**), which suggests that the disulfide bond formation may not be complete within the duration of the assay (**S9 Fig**).

As further validation of the HsFpn-PR73 structure, we next examined contributions from other binding site residues. We made alanine mutations to Gln194, Asp325, Tyr333, Thr501, and Phe508; and 2 disease-related mutations Tyr64Asn and Thr320Val. All the mutants have reduced binding affinity. Notably, Gln194Ala has the greatest effect with *K*_*D*_ approximately 6.11 μM (**Fig 6C** and **S2 Table**), indicating that the interaction between Gln194 and Ida(NHPal)11, which is also unique to PR73 (**Fig 5C**), plays a significant role in the high potency of PR73.

**Fig 7:**
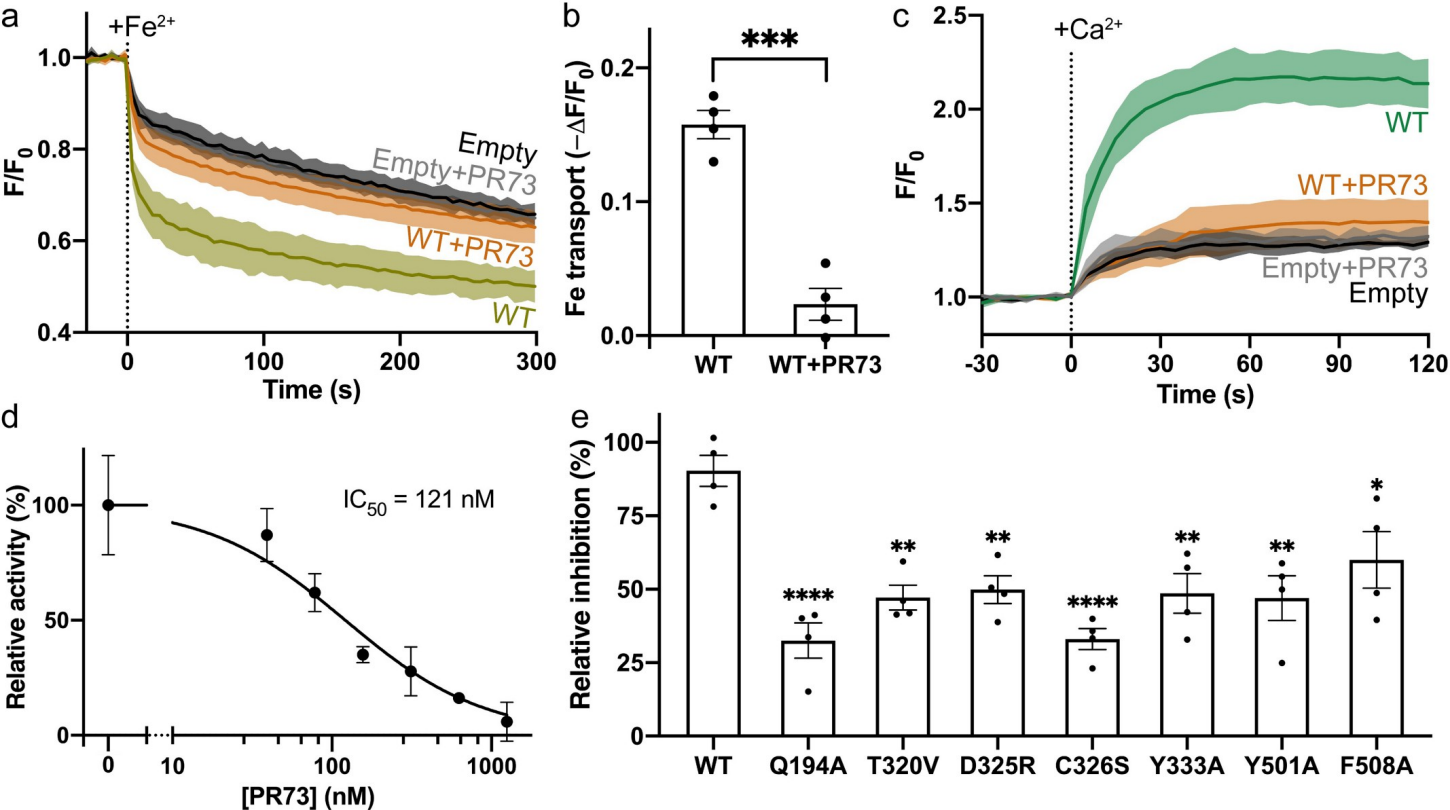
Inhibition of Fe^2+^ and Ca^2+^ transport by PR73. (**a**) Time-dependent Fe^2+^ uptake by HEK cells expressing WT Fpn. Approximately 100 μM Fe^2+^ (stabilized by 1 mM ascorbic acid) was administered at time zero. (**b**) Fpn-specific Fe^2+^ transport from data in (**a**). Each bar represents the change in fluorescence in WT after subtraction of that in empty vector control. Unpaired Student’s *t* test, *p* < 0.005. (**c**) Time-dependent Ca^2+^ uptake by HEK cells with or without HsFpn. Approximately 500 μM of Ca^2+^ was administered at time zero. The presence of 2 μM PR73 inhibits the transport. (**d**) Inhibition of Ca^2+^ transport by Fpn at different concentrations of PR73. (**e**) Relative inhibition of PR73 against HsFpn mutants. Dunnett’s test was used as a post hoc test following one-way analysis of variance with the WT as control. In (**a**) and (**c**), the solid lines represent the mean (*n* = 4) and the shaded areas the SD. In (**b**), (**d**), and (**e**), data are plotted as means with error bars representing the SEM. The solid black line in (**d**) is the fit of data to a single-binding site inhibition equation. Statistical significances are indicated: *, *p* < 0.05; **, *p* < 0.01; ***, *p* < 0.005; ****, *p* < 0.001. Source data for (**a**–**e**) can be found in **S1 Data**. HEK, human embryonic kidney; SEM, standard error of the mean; SD, standard deviation; WT, wild type.

We then measured inhibition of ion transport by PR73 in 3 cell-based transport assays. For the first, HsFpn was expressed in human embryonic kidney (HEK) cells (**S10 Fig**), and the cells were loaded with a Fe^2+^-sensitive dye (**Materials and methods**). In the second, cells were loaded with a pH-sensitive dye (**Materials and methods**), and the fluorescence change was initiated by the addition of Co^2+^ to the solution. Uptake of Co^2+^ by Fpn is accompanied by export of H^+^ [2], which leads to decreased intracellular pH. PR73 (2 μM) significantly reduces the transport activity in both assays (**Figs 7A, 7B,** and **S11)**. While there is a noticeable fluorescence change in the empty vector transfected cells, this change is significantly less than the specific transport when Fpn is expressed (**Figs 7A** and **S11A**) and likely due to nonspecific transport mediated by endogenous proteins in the cells. Lastly, the third assay measured Ca^2+^ uptake into HsFpn-expressing cells loaded with a Ca^2+^-sensitive dye (**Materials and methods**). We recently discovered that HsFpn can also function as a Ca^2+^ uniporter [24]. Because of the high background present in the Fe^2+^ and Co^2+^ transport assays, we used this Ca^2+^ transport assay to construct a dose-response profile for PR73 and found that PR73 inhibits Ca^2+^ transport with a half maximal inhibitory concentration (IC_50_) of approximately 121 nM (**Fig 7C** and **7D**). Each of the mutations used in the binding assay was also tested in the cell-based transport assay and exhibited significantly reduced relative inhibition by PR73 (**Figs 7E** and **S10**). Results from both the binding and transport assays are consistent with the structure of PR73-bound HsFpn.

## Discussion

Here, we report a study on 2 inhibitors of HsFpn. First, we showed that mouse monoclonal 11F9 Fab, which was developed to bind to a monkey homolog of Fpn, binds to HsFpn with nanomolar affinity and inhibits ion transport. We determined the structure of HsFpn in complex with 11F9 Fab and found that the Fab interacts with both the NTD and CTD of HsFpn from the intracellular side, and the interactions likely stabilize the transporter in an outward-facing conformation.

**Fig 8:**
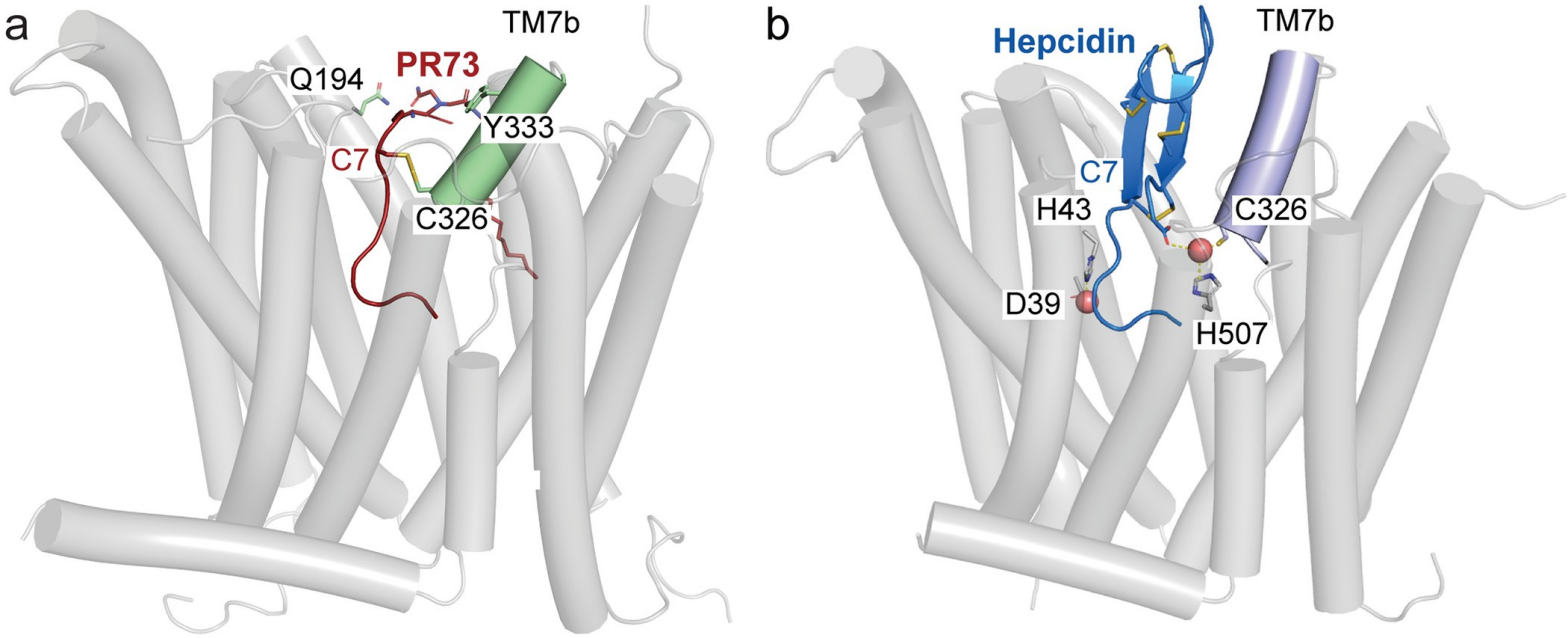
Structural comparison of PR73 and hepcidin-bound HsFpn. Side views of HsFpn-PR73 (**a**) and HsFpn-Hepcidin (6WIK) (**b**) highlight the disulfide bridge and metal ion coordination. In (**a**), TM7b is colored pale green and PR73 in brick red. In (**b**), TM7b is colored in light blue, hepcidin shown as marine cartoon with cysteine residues, and C-terminal carboxylate shown as sticks.

Second, we identified critical components of PR73-HsFpn interactions. We determined the structure of HsFpn in complex with PR73, and we find that the first 6 residues of PR73 assume a similar conformation to that of hepcidin, even though 3 of the 6 residues in PR73 are modified to unnatural amino acids, and that these residues interact with HsFpn similar to hepcidin. However, residues 7–11 of PR73 interact with HsFpn differently. While hepcidin has a compact and well-folded structure with 4 disulfide bridges, PR73 has none. The free Cys7 of PR73 could form a disulfide bond to Cys326 of HsFpn (**Fig 8A**), and we show that the interaction contributes significantly to the binding of PR73. Although it was proposed previously that hepcidin form a disulfide bridge with HsFpn, the structure of hepcidin-HsFpn shows that its internal cysteine bridges are intact (**Fig 8B**). The structure also shows that the flexibility of PR73 affords the interaction between residue 11 of PR73 and Gln194 of HsFpn, and we show that this interaction also contributes significantly to the binding. Finally, the structure shows that the hydrophobic acyl chain of the palmitic acid may extend into the hydrophobic core of the membrane to anchor PR73 in the membrane and thus may enhance its binding affinity.

Based on the structure of PR73 in complex with HsFpn, and considering that extracellular redox potential is usually in the range permissible for disulfide bridge formation, we think that Cys7 of PR73 would form a disulfide bridge with Cys326 of HsFpn, which could significantly enhance the inhibition. Likewise, the stability of hepcidin is also dependent on formation of stable disulfide bridges and we surmise that under normal physiological conditions, hepcidin is well folded and optimized to inhibit HsFpn. If macrophages or hepatocytes encounter conditions that do not favor stable disulfide bridge formation, then inhibition of Fpn by either hepcidin or PR73 would be compromised.

Minihepcidins are promising therapeutic reagents being pursued for the treatment of human diseases like β-thalassemia and hemochromatosis [16,18]. Our study provides a structural framework that highlights unique interactions between PR73 and Fpn and may facilitate and guide future drug development targeting HsFpn.

## Materials and methods

### Cloning, expression, and purification of HsFpn

The cDNA of HsFpn (UniProt ID: Q9NP59) was codon optimized, synthesized, and cloned into a pFastBac dual vector. A tobacco etch virus (TEV) protease site and an octa-histidine (8×His) tag were added to the C-terminus of the protein. The Back-to-Bac method (Invitrogen) was used to express HsFpn was expressed in Sf9 (*Spodoptera frugiperda*). Purification of HsFpn follows the same protocol reported for TsFpn [2]. Size exclusion chromatography (SEC) was used to collect the purified HsFpn in FPLC buffer consisting of 20 mM HEPES (pH7.5), 150 mM NaCl, and 1 mM (w/v) n-dodecyl-β-D-maltoside (DDM, Anatrace). The Quikchange method (stratagene) was used to generate HsFpn mutations. Mutations were verified by sequencing. Mutant HsFpn proteins were expressed and purified following the same protocol for the WT.

### Octet biolayer interferometry

Biolayer interferometry (BLI) assays were performed at 30°C under constant shaking at 1,000 rpm using an Octet system (FortéBio). First, amine-reactive second-generation (AR2G) biosensor (Sartorius) tips were activated in 20 mM 1-ethyl-3-[3-dimethylaminopropyl]carbodiimide hydrochloride (EDC) and 10 mM N-hydroxysulfosuccinimide (Sulfo-NHS) for 300 s. Then, the tips were immobilized with 5 μg/mL of 11F9 Fab in the FPLC buffer for 600 s. The tips were quenched in 1 M ethanolamine (pH 8.5) for 300 s. The tips with immobilized ligands were equilibrated in the FPLC buffer for 120 s and transferred to wells with a concentration gradient of HsFpn (400, 200, 100, 50, and 25 nM) for 300 s (association) and returned to the equilibration wells for dissociation (300 s). To measure PR73 binding, the tips were immobilized with Fpn at a concentration of 2 μg/mL in the FPLC buffer for 600 s. After quenching the immobilization reaction, the tips were transferred to wells with a concentration gradient of PR73 (10, 5, 2.5, 1.25 μM) for 300 s (association) and back to equilibration wells for 300 s (dissociation). Binding curves were aligned and corrected with the channel of no analyst protein. The association and disassociation phases were fit with 1-exponential functions to extract association rate constant *k*_*a*_ and dissociation rate constant *k*_*d*_ of the binding, which were used to calculate the dissociation constant *K*_D_.

### Reconstitution of Fpn into liposomes

The 1-palmitoyl-2-oleoyl-sn-glycero-3-phosphoethanolamine (POPE, Avanti Polar Lipids) and 1-palmitoyl-2-oleoyl-sn-glycero-3-phospho-(1’-rac)-glycerol (POPG, Avanti Polar Lipids) was mixed at 3:1 molar ratio, dried with Argon, and vacuumed overnight to remove chloroform. The lipid was resuspended in reconstitution buffer (20 mM HEPES (pH 7.5), 100 mM NaCl) to a final concentration of 10 mg/mL. The lipid was sonicated until it appeared transparent. A total of 40 mM n-decyl-β-D-maltoside (DM, Anatrace) was added and the sample was incubated for 2 h at room temperature under gentle agitation. HsFpn was added at a 1:100 (w/w, protein:lipid) ratio. Dialysis was performed at 4°C with the reconstitution buffer to remove the detergent. The dialysis buffer was changed daily for 3 days and then harvested on day 4. Liposome samples were aliquoted and frozen at −80°C for future use.

### Co^2+^ and Fe^2+^ flux assays in proteoliposomes

Proteoliposome samples of HsFpn were mixed with 250 μM calcein, with or without 20 μM 11F9 Fab, and underwent 3 cycles of freeze-thaw. The liposomes were extruded to homogeneity with a 400 nm filter (NanoSizer Extruder, T&T Scientific Corporation). Excess calcein was removed with a desalting column (PD-10, GE Healthcare) that had been equilibrated with the dialysis buffer. A quartz cuvette was used to detect the fluorescence at 37°C. For the samples loaded with the Fab, an additional 20 μM of 11F9 Fab was incubated with the liposomes prior to reading. The cuvette was read at 10 s intervals with 494 nm excitation and 513 nm emission in a FluoroMax-4 spectrofluorometer (HORIBA). Approximately 100 μM CoCl_2_ or 100 μM Fe^2+^ (together with 1 mM ascorbate-Na) was added to initiate transport.

### Preparation of Fpn-11F9 complex in nanodisc

An established protocol [25] was used to express and purify membrane scaffold protein (MSP) 1D1. Lipid preparation was carried out by mixing 1-palmitoyl-2-oleoyl-sn-glycero-3-phosphocholine (POPC, Avanti Polar Lipids), POPE, and POPG at a molar ratio of 3:1:1. The lipid mixture was dried with Argon and vacuumed for 2 h. The lipid was resuspended with 14 mM DDM [26]. HsFpn, MSP1D1, and the lipid mixture were mixed at a molar ratio of 1:2.5:50 and incubated on ice for 1 h for nanodisc reconstitution. A total of 60 mg/mL of Biobeads SM2 (Bio-Rad) were added 3 times within 3 h to remove detergents. After the samples were incubated with the Biobeads overnight at 4°C, the Biobeads were removed. 11F9 Fab was added to the nanodisc sample at a molar ratio of 1.1:1 to Fpn. The complex was incubated on ice for 30 min before it was loaded onto a SEC column that had been equilibrated with 20 mM HEPES (pH 7.5) and 150 mM NaCl. The purified nanodisc sample was concentrated to 10 mg/ml and incubated with 10 mM CoCl_2_ or 1 mM PR73 for 30 min before cryo-EM grid preparation.

### Cryo-EM sample preparation and data collection

The cryo-EM grids were prepared with Thermo Fisher Vitrobot Mark IV. The Quantifoil R1.2/1.3 Cu grids were glow-discharged with air at 10 mA for 15 s using Plasma Cleaner (PELCO EasiGlow). Aliquots of 3.5 μL of the nanodisc sample were applied to the glow-discharged grids. The grids were blotted with filter paper (Ted Pella) for 4.0 s and plunged into liquid ethane cooled with liquid nitrogen. A total of 4,251 (for Co^2+^-bound Fpn) and 4,941 (for PR73-bound Fpn) micrograph stacks were collected on a Titan Krios at 300 kV equipped with a K3 Summit direct electron detector (Gatan) and a Quantum energy filter (Gatan) at a nominal magnification of 81,000× and defocus values from −2.25 to −1.0 μM (HsFpn-Co^2+^) or −2.5 to −0.8 μM (HsFpn-PR73). Each stack was exposed for 0.0875 s per frame in the super-resolution mode for a total of 40 frames per stack, which results in a total dose of approximately 50 e^-^/Å^2^. The stacks were motion corrected with MotionCor2 [27] and binned 2-fold. The final pixel size is 1.08 Å/pixel (HsFpn-Co^2+^) or 1.10 Å/pixel (HsFpn-PR73). In the meantime, dose weighting was performed [28]. The defocus values were estimated with Gctf [29].

### Cryo-EM data processing

A total of 2,175,353 (HsFpn-Co^2+^) and 2,960,056 (HsFpn-PR73) particles were automatically picked based on a reference map of TsFpn-11F9 (EMD-21460) that was low-pass filtered to 20 Å in RELION 3.1 [30–32]. Particles were extracted and imported into CryoSparc [33] for 2D classification. A total of 1,242,825 (HsFpn-Co^2+^) and 918,469 particles (HsFpn-PR73) were selected from good classes in 2D classification. A total of 100,000 particles for the good classes were used to generate 4 initial reference models. Multiple rounds of heterogeneous refinement were performed with particles selected from the 2D classification until <5% input particles were classified into bad classes. A total of 215,164 particles (HsFpn-Co^2+^) or 454,601 particles (HsFpn-PR73) were subjected to non-uniform (NU) refinement. After handedness correction, local refinement and CTF refinement were performed, resulting in a reconstruction with an overall resolution of 3.0 Å for HsFpn-Co^2+^ and 2.6 Å for HsFpn-PR73. Additional rounds of heterogenous refinement were performed for HsFpn-PR73 with 3 reference models of “class similarity” of 1 to further improve the density for PR73. Classes with strong densities for PR73 were selected and subjected to NU refinement. The final total of 162,586 particles yielded a reconstruction with an overall resolution of 2.7 Å for HsFpn-PR73. Resolution was estimated with the gold-standard Fourier shell correlation 0.143 criterion [34]. Local resolution of the maps was estimated in CryoSparc [33].

### Model building and refinement

The structure of apo HsFpn (PDB ID 6W4S) and the 11F9 Fab (from PDB ID 6VYH) were individually docked into density maps in Chimera [35]. The docked model was manually adjusted in COOT [36]. PHENIX [37] was used for real space refinements with secondary structure and geometry restraints. The EMRinger Score [38] was calculated for the models. Structure figures were prepared in Pymol and ChimeraX [39].

### Fe^2+^, H^+^, and Ca^2+^ transport assays in HEK cells

The pEG BacMam plasmids with WT or mutant Fpn or the empty plasmid were transfected into HEK 293S cells on black wall 96-well microplates coated with poly-D-lysine (Invitrogen/Thermo Fisher). After 2 days, cells were washed in the live cell imaging solution (LCIS) containing 20 mM HEPES (pH 7.4), 140 mM NaCl, 2.5 mM KCl, 1.0 mM MgCl_2_, and 5 mM D-glucose. The loading of PhenGreen FL (Invitrogen/Thermo Fisher, Diacetate) for Fe^2+^ transport, pHrodo Red (Invitrogen/Thermo Fisher, AM) for H^+^ transport, or Fluo-4 (Invitrogen/Thermo Fisher, AM, cell-permeant) for Ca^2+^ transport was performed following the manufacturer’s protocols. After the dye loading finished, free dyes were washed away, and cells in each well were maintained in 90 μL LCIS. All transport assays were performed in the FlexStation 3 Multi-Mode Microplate Reader (Molecular Devices) at 37°C. Fluorescence changes were recorded at an excitation and emission wavelength of 492 nm and 517 nm for Fe^2+^ transport, 544 nm and 590 nm for H^+^ transport, or 485 nm and 538 nm for Ca^2+^ transport with 5 s intervals. For PR73 inhibition, cells were incubated with desired concentrations of PR73 for 5 min prior to reading. Transport was initiated by the addition of 10 μL freshly prepared ligand stock solution to achieve 100 μM Fe^2+^ (together with 1 mM ascorbate-Na) or 500 μM Co^2+^ or Ca^2+^. For Fe^2+^ and H^+^ transport, relative fluorescence changes at the equilibrium stage were averaged to represent intracellular [Fe^2+^] or pH changes. For Ca^2+^ uptake, the slopes of straight lines fitted to transport data within 25 s were used to represent initial rates.

## Supporting information

S1 Fig Reconstitution and characterization of HsFpn-11F9 complex. Related to Fig 2. (**a**) Left: structural alignment of HsFpn (grey, PDB ID 6W4S) and TsFpn (teal, PDB ID 6VYH). Right: the intercellular side of TsFpn is shown as a grey surface with the epitope of 11F9, defined as residues within 4 Å from 11F9, colored in magenta. (**b**) Binding of 11F9 Fab to HsFpn measured by Octet BLI. (**c**) Size-exclusion chromatography profiles and SDS-PAGE gel image (inset) of HsFpn-11F9 in nanodisc. HsFpn-11F9 complex (solid line) eluted significantly earlier than HsFpn alone (dotted line). The later peak from the complex sample comes from the Fab in excess. (**d**) Co^2+^ import into proteoliposomes measured by fluorescent changes (F/F_0_) of a transition metal ion-sensitive dye (calcein). Addition of the Fab to the inside and outside of the liposome inhibits HsFpn transport activity. Approximately 100 μM Co^2+^ was added at time zero. (**e**) Initial rates of fluorescence change with and without the Fab. A scatter plot is overlaid on each bar. Unpaired Student’s *t* test, *p* = 0.0013. (**f**) Fe^2+^ influx and inhibition by the Fab in proteoliposomes. Approximately 100 μM Fe^2+^ was added at time zero. (**g**) Initial rates of fluorescence change with and without the Fab. Unpaired Student’s *t* test, *p* = 0.0082. In (**d**) and (**f**), traces are shown as solid lines (mean) with shaded regions (SD) from at least 3 biological repeats (n = 3). In (**e**) and (**g**), data are plotted as means with error bars representing the SEM. Statistical significances are indicated: **, *p* < 0.01; ***, *p* < 0.005. Titration of Fe^2+^ (**h**) and Co^2+^ (**i**) into 125 *μ*M of free calcein dye at 37°C. The concentrations of Fe^2+^ and Co^2+^ have similar linear relationships of F/F_0_ within the first 15 *μ*M. (**j**) Structural comparison between apo (PDB ID 6W4S) and Co^2+^-bound HsFpn. Notice that the Fab used in 6W4S for structural determination binds to the extracellular side and interacts only with the NTD of HsFpn. Source data for (**b**–**g**) can be found in **S1 Data**.
(TIF)

S2 Fig Cryo-EM analysis of HsFpn-Co2+ in nanodisc. Related to Fig 2. (**a**) Representative electron micrograph (upper panel) and 2D class averages (lower panel). (**b**) Workflow of data processing for single-particle reconstruction. (**c**) The gold-standard Fourier shell correlation (FSC) curves for the final map (left panel) and map-to-model FSC curves (right panel). (**d**) Direction distribution of particles used in the final 3D reconstruction. (**e**) Local resolution map colored from 2.4 Å (blue) to >4.0 Å (red). Source data for (**c**) can be found in **S1 Data**.
(TIF)

S3 Fig Cryo-EM densities of TM helices and AHs. Related to Fig 2. Densities for HsFpn-Co^2+^ are shown as pink mesh. Residues within the ranges indicated below are rendered in stick representations and colored in pale green for NTD and light orange for CTD.
(TIF)

S4 Fig Cryo-EM analysis of HsFpn-PR73 in nanodisc. Related to Fig 3. (**a**) Representative electron micrograph (upper panel) and 2D class averages (lower panel). (**b**) Workflow of data processing for single-particle reconstruction. (**c**) The gold-standard Fourier shell correlation (FSC) curves for the final map (left panel) and map-to-model FSC curves (right panel). (**d**) Direction distribution of particles used in the final 3D reconstruction. (**e**) Local resolution map colored from 2.4 Å (blue) to >4.0 Å (red). Source data for (**c**) can be found in **S1 Data**.
(TIF)

S5 Fig Cryo-EM densities of TM helices, AHs, and PR73 in HsFpn-PR73. Related to Fig 3. Densities of HsFpn-PR73 are shown as blue mesh. Residues within the ranges indicated below are represented as sticks and colored in pale green, light orange, or brick red for the NTD, CTD, and PR73, respectively.
(TIF)

S6 Fig Lipid molecules bound to HsFpn. Related to Fig 3. Side view of 2 phospholipids (shown as spheres) located on the intracellular side in the HsFpn-PR73 structure (middle panel). Interactions of these lipids with residues of HsFpn (left and right panels). Side chains of residues within 4 Å from the lipids are shown as sticks. Hydrophilic interactions are indicated with yellow dashed lines. Densities of the lipids are contoured at 3.5σ as blue mesh.
(TIF)

S7 Fig Binding site interactions PR73 vs. hepcidin. Related to Fig 5. Ligplot of hepcidin (**a** and **c**) and PR73 (**b** and **d**) showing interactions in the binding pocket of HsFpn. The peptide is shown as purple sticks. Interactions common in both structures are marked by red circles.
(TIF)

S8 Fig Native disulfide bridges in the HsFpn-PR73 Fab structure. **Related to Fig 5.** (**a**) Cys553 near the C-terminus of HsFpn forms a disulfide bond with Cys367 between TM8 and TM9. Disulfide bonds in the light chain (**b**) and heavy chain (**c**) of the Fab. All density maps are contoured at 4.5σ as blue mesh.
(TIF)

S9 Fig Incubation length effect on PR73 dissociation from HsFpn. **Related to Fig 6. (a)** Dissociation phase of BLI assay is shown as mean (solid black line) and SD (gray shadow) at each point. The fit to data is shown as a colored curve of red, yellow, and green for incubation time of 10, 60, and 120 min, respectively. **(b)** Dissociation rate constants (*k*_*d*_) of HsFpn to PR73 with different lengths of incubation time. The height of the bar graphs represents the mean of at least 3 measurements and the error bar SEM. Source data for (**b**) can be found in **S1 Data**.
(TIF)

S10 Fig Expression of WT and mutant Fpn in HEK cells assessed by western blot. Related to Fig 7.
(TIF)

S11 Fig Inhibition of PR73 against the Co2+ transport by Fpn. **Related to Fig 7.** (**a**) Co^2+^ import-induced H^+^ export by Fpn in HEK cells indicated by fluorescence change (F/F_0_) of a pH-sensitive dye (pHrodo Red) loaded inside cells. A total of 500 μM of Co^2+^ was administered at time zero. The solid lines represent the mean of 4 repeats and the shaded areas the SD. (**b**) Fpn-specific pH changes from data in (**a**). Each bar represents the change in fluorescence after subtraction of the fluorescence change in empty vector control cells. Data is plotted as means (*n* = 3) with error bars representing the SEM. Unpaired Student’s *t* test, *p* = 0.0052. Source data for (**a**–**b**) can be found in **S1 Data**.
(TIF)

S1 Table Summary of cryo-EM data collection, processing, and refinement. (DOCX)

S2 Table Binding affinities of PR73 to Fpn measured by Octet BLI. Related to Fig 6.
(DOCX)

S1 Data Source data for graphs in this paper. (XLSX)

S1 Raw image Uncropped western blotting and SDS-PAGE gel image in this paper. (PDF)

## Abbreviations

AH: amphipathic helix
BLI: biolayer interferometry
BME: β-mercaptoethanol
CTD: C-terminal domain
cryo-EM: cryo-electron microscopy
Fab: fragment of antigen binding
Fpn: ferroportin
HEK: human embryonic kidney
LCIS: live cell imaging solution
MSP: membrane scaffold protein
NTD: N-terminal domain
NU: non-uniform
RMSD: root-mean-squared distance
SEC: size exclusion chromatography
TEV: tobacco etch virus
TM: transmembrane
WT: wild type

## References

[1] Ganz T. Hepcidin—a regulator of intestinal iron absorption and iron recycling by macrophages. Best Pract Res Clin Haematol. 2005 Jun;18(2):171–82. doi: 10.1016/j.beha.2004.08.020 15737883

[2] Pan Y, Ren Z, Gao S, Shen J, Wang L, Xu Z, et al. Structural basis of ion transport and inhibition in ferroportin. Nat Commun. 2020 Nov 10;11:5686. doi: 10.1038/s41467-020-19458-6 33173040 PMC7655804

[3] Vlasveld LT, Janssen R, Bardou-Jacquet E, Venselaar H, Hamdi-Roze H, Drakesmith H, et al. Twenty Years of Ferroportin Disease: A Review or An Update of Published Clinical, Biochemical, Molecular, and Functional Features. Pharmaceuticals (Basel). 2019 Sep 9;12(3):E132. doi: 10.3390/ph12030132 31505869 PMC6789780

[4] Donovan A, Lima CA, Pinkus JL, Pinkus GS, Zon LI, Robine S, et al. The iron exporter ferroportin/Slc40a1 is essential for iron homeostasis. Cell Metab. 2005 Mar;1(3):191–200. doi: 10.1016/j.cmet.2005.01.003 16054062

[5] Aschemeyer S, Qiao B, Stefanova D, Valore EV, Sek AC, Ruwe TA, et al. Structure-function analysis of ferroportin defines the binding site and an alternative mechanism of action of hepcidin. Blood. 2018 Feb 22;131(8):899–910. doi: 10.1182/blood-2017-05-786590 29237594 PMC5824336

[6] Nemeth E, Tuttle MS, Powelson J, Vaughn MB, Donovan A, Ward DM, et al. Hepcidin regulates cellular iron efflux by binding to ferroportin and inducing its internalization. Science. 2004 Dec 17;306(5704):2090–3. doi: 10.1126/science.1104742 15514116

[7] Babitt JL, Huang FW, Xia Y, Sidis Y, Andrews NC, Lin HY. Modulation of bone morphogenetic protein signaling in vivo regulates systemic iron balance. J Clin Invest. 2007 Jul;117(7):1933–9. doi: 10.1172/JCI31342 17607365 PMC1904317

[8] Truksa J, Peng H, Lee P, Beutler E. Bone morphogenetic proteins 2, 4, and 9 stimulate murine hepcidin 1 expression independently of Hfe, transferrin receptor 2 (Tfr2), and IL-6. Proc Natl Acad Sci U S A. 2006 Jul 5;103(27):10289–93. doi: 10.1073/pnas.0603124103 16801541 PMC1502450

[9] Drakesmith H, Nemeth E, Ganz T. Ironing out ferroportin. Cell Metab. 2015 Nov 3;22(5):777–87. doi: 10.1016/j.cmet.2015.09.006 26437604 PMC4635047

[10] Pietrangelo A. Ferroportin disease: pathogenesis, diagnosis and treatment. Haematologica. 2017 Dec;102(12):1972–84. doi: 10.3324/haematol.2017.170720 29101207 PMC5709096

[11] Ginzburg YZ. Hepcidin-ferroportin axis in health and disease. Vitam Horm. 2019;110:17–45. doi: 10.1016/bs.vh.2019.01.002 30798811 PMC7730607

[12] Billesbølle CB, Azumaya CM, Kretsch RC, Powers AS, Gonen S, Schneider S, et al. Structure of hepcidin-bound ferroportin reveals iron homeostatic mechanisms. Nature. 2020 Oct;586(7831):807–11. doi: 10.1038/s41586-020-2668-z 32814342 PMC7906036

[13] Park CH, Valore EV, Waring AJ, Ganz T. Hepcidin, a urinary antimicrobial peptide synthesized in the liver. J Biol Chem. 2001 Mar 16;276(11):7806–10. doi: 10.1074/jbc.M008922200 11113131

[14] Hunter HN, Fulton DB, Ganz T, Vogel HJ. The solution structure of human hepcidin, a peptide hormone with antimicrobial activity that is involved in iron uptake and hereditary hemochromatosis. J Biol Chem. 2002 Oct 4;277(40):37597–603. doi: 10.1074/jbc.M205305200 12138110

[15] Jordan JB, Poppe L, Haniu M, Arvedson T, Syed R, Li V, et al. Hepcidin revisited, disulfide connectivity, dynamics, and structure. J Biol Chem. 2009 Sep 4;284(36):24155–67. doi: 10.1074/jbc.M109.017764 19553669 PMC2782009

[16] Preza GC, Ruchala P, Pinon R, Ramos E, Qiao B, Peralta MA, et al. Minihepcidins are rationally designed small peptides that mimic hepcidin activity in mice and may be useful for the treatment of iron overload. J Clin Invest. 2011 Dec;121(12):4880–8. doi: 10.1172/JCI57693 22045566 PMC3225996

[17] Fernandes A, Preza GC, Phung Y, De Domenico I, Kaplan J, Ganz T, et al. The molecular basis of hepcidin-resistant hereditary hemochromatosis. Blood. 2009 Jul 9;114(2):437–43. doi: 10.1182/blood-2008-03-146134 19383972 PMC2714214

[18] Ramos E, Ruchala P, Goodnough JB, Kautz L, Preza GC, Nemeth E, et al. Minihepcidins prevent iron overload in a hepcidin-deficient mouse model of severe hemochromatosis. Blood. 2012 Nov 1;120(18):3829–36. doi: 10.1182/blood-2012-07-440743 22990014 PMC3488893

[19] Ganz T. Hepcidin and its role in regulating systemic iron metabolism. Hematology Am Soc Hematol Educ Program. 2006;29–35:507. doi: 10.1182/asheducation-2006.1.29 17124036

[20] Chua K, Fung E, Micewicz ED, Ganz T, Nemeth E, Ruchala P. Small cyclic agonists of iron regulatory hormone hepcidin. Bioorg Med Chem Lett. 2015 Nov 1;25(21):4961–9. doi: 10.1016/j.bmcl.2015.03.012 25813158 PMC4567957

[21] Fung E, Chua K, Ganz T, Nemeth E, Ruchala P. Thiol-derivatized minihepcidins retain biological activity. Bioorg Med Chem Lett. 2015 Feb 15;25(4):763–6. doi: 10.1016/j.bmcl.2014.12.094 25599838 PMC4318710

[22] Mitchell CJ, Shawki A, Ganz T, Nemeth E, Mackenzie B. Functional properties of human ferroportin, a cellular iron exporter reactive also with cobalt and zinc. Am J Physiol Cell Physiol. 2014 Mar 1;306(5):C450–459. doi: 10.1152/ajpcell.00348.2013 24304836 PMC4042619

[23] Morrissey J, Baxter IR, Lee J, Li L, Lahner B, Grotz N, et al. The ferroportin metal efflux proteins function in iron and cobalt homeostasis in Arabidopsis. Plant Cell. 2009 Oct;21(10):3326–38. doi: 10.1105/tpc.109.069401 19861554 PMC2782287

[24] Shen J, Wilbon AS, Zhou M, Pan Y. (2023) Mechanism of Ca2+ transport by ferroportin. eLife 12:e82947. doi: 10.7554/eLife.82947 36648329 PMC9883014

[25] Martens C, Stein RA, Masureel M, Roth A, Mishra S, Dawaliby R, et al. Lipids modulate the conformational dynamics of a secondary multidrug transporter. Nat Struct Mol Biol. 2016 Aug;23(8):744–51. doi: 10.1038/nsmb.3262 27399258 PMC5248563

[26] Autzen HE, Myasnikov AG, Campbell MG, Asarnow D, Julius D, Cheng Y. Structure of the human TRPM4 ion channel in a lipid nanodisc. Science. 2018 Jan 12;359(6372):228–32. doi: 10.1126/science.aar4510 29217581 PMC5898196

[27] Zheng SQ, Palovcak E, Armache JP, Verba KA, Cheng Y, Agard DA. MotionCor2: anisotropic correction of beam-induced motion for improved cryo-electron microscopy. Nat Methods. 2017 Apr;14(4):331–2. doi: 10.1038/nmeth.4193 28250466 PMC5494038

[28] Grant T, Grigorieff N. Measuring the optimal exposure for single particle cryo-EM using a 2.6 Å reconstruction of rotavirus VP6. Elife. 2015 May 29;4:e06980. 26023829 10.7554/eLife.06980 PMC4471936

[29] Zhang K. Gctf: Real-time CTF determination and correction. J Struct Biol. 2016 Jan;193(1):1–12. doi: 10.1016/j.jsb.2015.11.003 26592709 PMC4711343

[30] Kimanius D, Forsberg BO, Scheres SH, Lindahl E. Accelerated cryo-EM structure determination with parallelisation using GPUs in RELION-2. Elife. 2016;15(5):e18722. doi: 10.7554/eLife.18722 27845625 PMC5310839

[31] Scheres SHW. RELION: implementation of a Bayesian approach to cryo-EM structure determination. J Struct Biol. 2012 Dec;180(3):519–30. doi: 10.1016/j.jsb.2012.09.006 23000701 PMC3690530

[32] Zivanov J, Nakane T, Forsberg BO, Kimanius D, Hagen WJ, Lindahl E, et al. New tools for automated high-resolution cryo-EM structure determination in RELION-3. Elife. 2018;9(7):e42166. doi: 10.7554/eLife.42166 30412051 PMC6250425

[33] Punjani A, Rubinstein JL, Fleet DJ, Brubaker MA. cryoSPARC: algorithms for rapid unsupervised cryo-EM structure determination. Nat Methods. 2017 Mar;14(3):290–6. doi: 10.1038/nmeth.4169 28165473

[34] Rosenthal PB, Henderson R. Optimal determination of particle orientation, absolute hand, and contrast loss in single-particle electron cryomicroscopy. J Mol Biol. 2003 Oct 31;333(4):721–45. doi: 10.1016/j.jmb.2003.07.013 14568533

[35] Pettersen EF, Goddard TD, Huang CC, Couch GS, Greenblatt DM, Meng EC, et al. UCSF Chimera—a visualization system for exploratory research and analysis. J Comput Chem. 2004 Oct;25(13):1605–12. doi: 10.1002/jcc.20084 15264254

[36] Emsley P, Lohkamp B, Scott WG, Cowtan K. Features and development of Coot. Acta Crystallogr D Biol Crystallogr. 2010 Apr;66(Pt 4):486–501. doi: 10.1107/S0907444910007493 20383002 PMC2852313

[37] Adams PD, Afonine PV, Bunkóczi G, Chen VB, Davis IW, Echols N, et al. PHENIX: a comprehensive Python-based system for macromolecular structure solution. Acta Crystallogr D Biol Crystallogr. 2010 Feb;66(Pt 2):213–21. doi: 10.1107/S0907444909052925 20124702 PMC2815670

[38] Barad BA, Echols N, Wang RYR, Cheng Y, DiMaio F, Adams PD, et al. EMRinger: side chain-directed model and map validation for 3D cryo-electron microscopy. Nat Methods. 2015 Oct;12(10):943–6. doi: 10.1038/nmeth.3541 26280328 PMC4589481

[39] Pettersen EF, Goddard TD, Huang CC, Meng EC, Couch GS, Croll TI, et al. UCSF ChimeraX: Structure visualization for researchers, educators, and developers. Protein Sci. 2021 Jan;30(1):70–82. doi: 10.1002/pro.3943 32881101 PMC7737788

