# Supplementary figures and images for: Structural basis of ferroportin inhibition by minihepcidin PR73
Source: PLoS Biol. 2023 Jan 17;21(1):e3001936. doi: 10.1371/journal.pbio.3001936 (PMC9882908; doi:10.1371/journal.pbio.3001936)

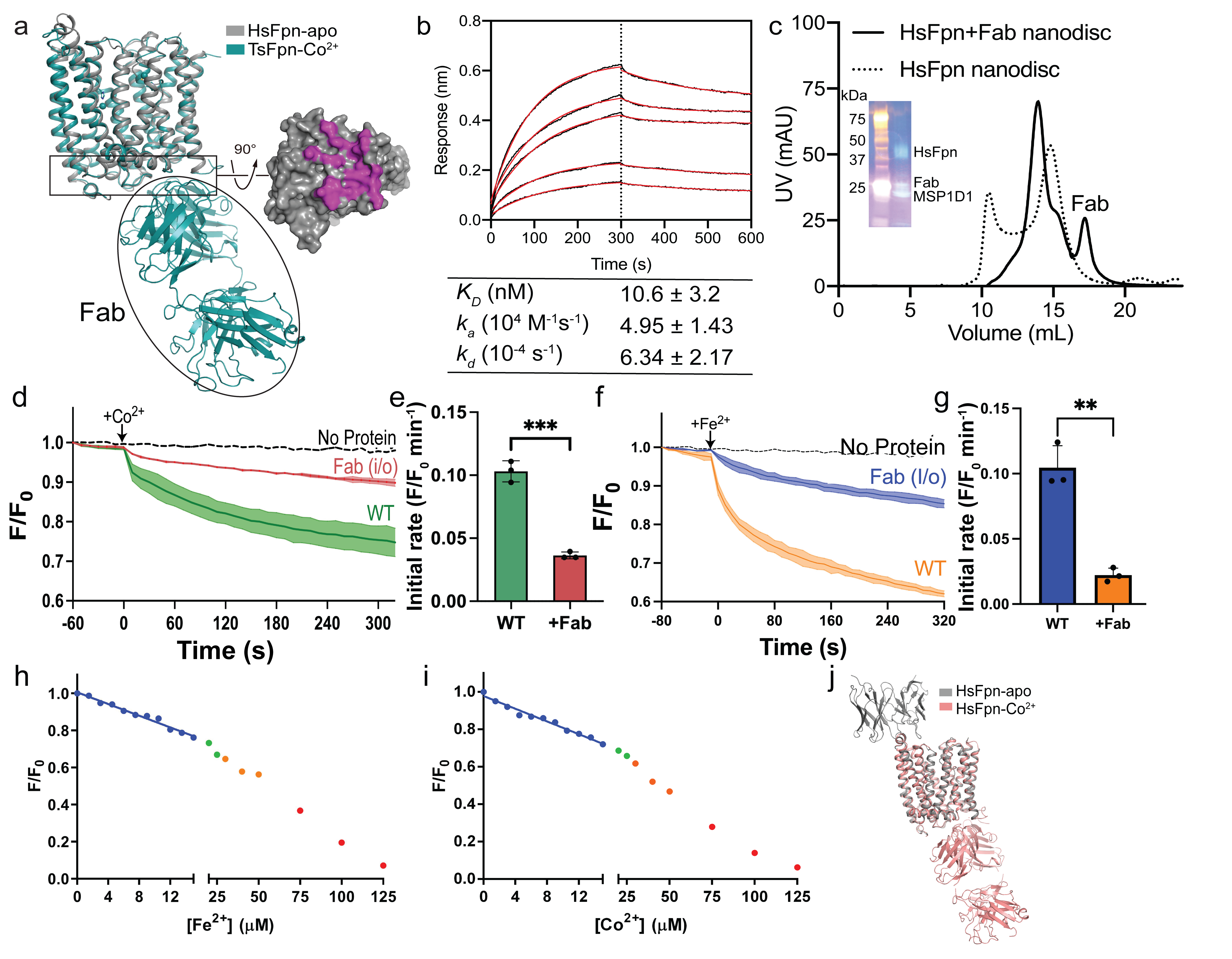

Supplement: S1 Fig — Related to Fig 2. (a) Left: structural alignment of HsFpn (grey, PDB ID 6W4S) and TsFpn (teal, PDB ID 6VYH). Right: the intercellular side of TsFpn is shown as a grey surface with the epitope of 11F9, defined as residues within 4 Å from 11F9, colored in magenta. (b) Binding of 11F9 Fab to HsFpn measured by Octet BLI. (c) Size-exclusion chromatography profiles and SDS-PAGE gel image (inset) of HsFpn-11F9 in nanodisc. HsFpn-11F9 complex (solid line) eluted significantly earlier than HsFpn alone (dotted line). The later peak from the complex sample comes from the Fab in excess. (d) Co2+ import into proteoliposomes measured by fluorescent changes (F/F0) of a transition metal ion-sensitive dye (calcein). Addition of the Fab to the inside and outside of the liposome inhibits HsFpn transport activity. Approximately 100 μM Co2+ was added at time zero. (e) Initial rates of fluorescence change with and without the Fab. A scatter plot is overlaid on each bar. Unpaired Student’s t test, p = 0.0013. (f) Fe2+ influx and inhibition by the Fab in proteoliposomes. Approximately 100 μM Fe2+ was added at time zero. (g) Initial rates of fluorescence change with and without the Fab. Unpaired Student’s t test, p = 0.0082. In (d) and (f), traces are shown as solid lines (mean) with shaded regions (SD) from at least 3 biological repeats (n = 3). In (e) and (g), data are plotted as means with error bars representing the SEM. Statistical significances are indicated: **, p < 0.01; ***, p < 0.005. Titration of Fe2+ (h) and Co2+ (i) into 125 μM of free calcein dye at 37°C. The concentrations of Fe2+ and Co2+ have similar linear relationships of F/F0 within the first 15 μM. (j) Structural comparison between apo (PDB ID 6W4S) and Co2+-bound HsFpn. Notice that the Fab used in 6W4S for structural determination binds to the extracellular side and interacts only with the NTD of HsFpn. Source data for (b–g) can be found in S1 Data. (TIF) [file pbio.3001936.s001.tif]

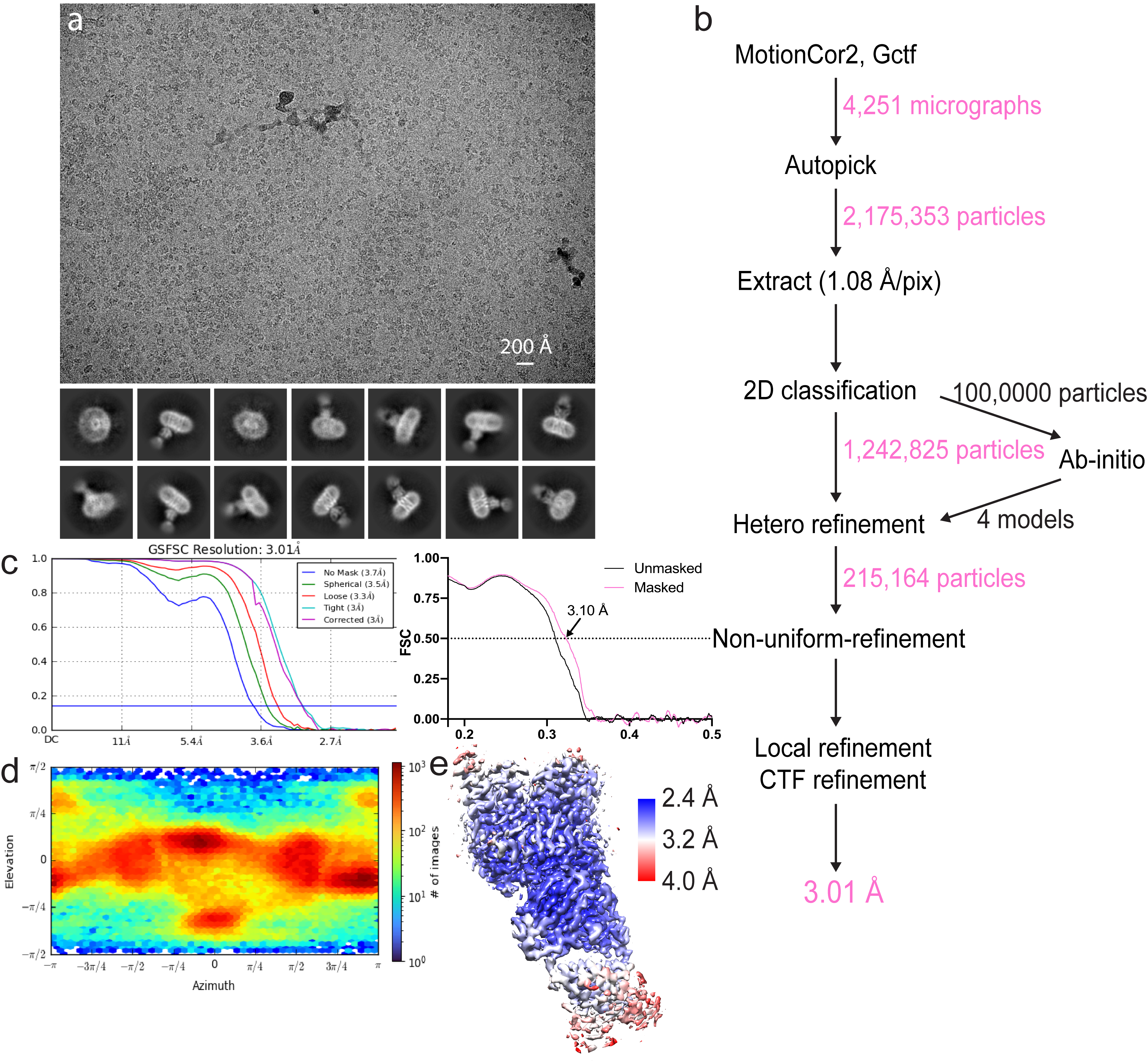

Supplement: S2 Fig — Related to Fig 2. (a) Representative electron micrograph (upper panel) and 2D class averages (lower panel). (b) Workflow of data processing for single-particle reconstruction. (c) The gold-standard Fourier shell correlation (FSC) curves for the final map (left panel) and map-to-model FSC curves (right panel). (d) Direction distribution of particles used in the final 3D reconstruction. (e) Local resolution map colored from 2.4 Å (blue) to >4.0 Å (red). Source data for (c) can be found in S1 Data. (TIF) [file pbio.3001936.s002.tif]

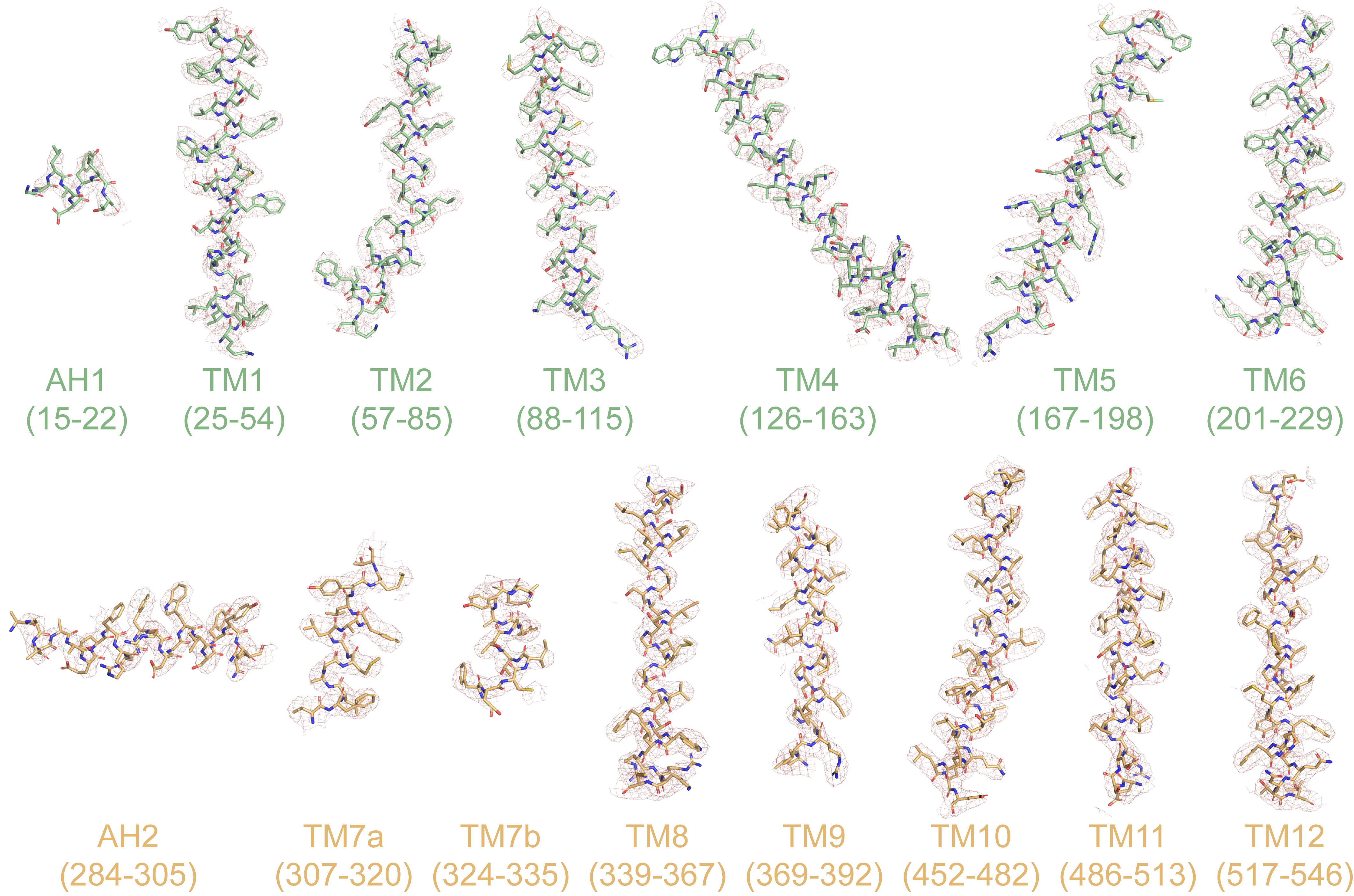

Supplement: S3 Fig — Related to Fig 2. Densities for HsFpn-Co2+ are shown as pink mesh. Residues within the ranges indicated below are rendered in stick representations and colored in pale green for NTD and light orange for CTD. (TIF) [file pbio.3001936.s003.tif]

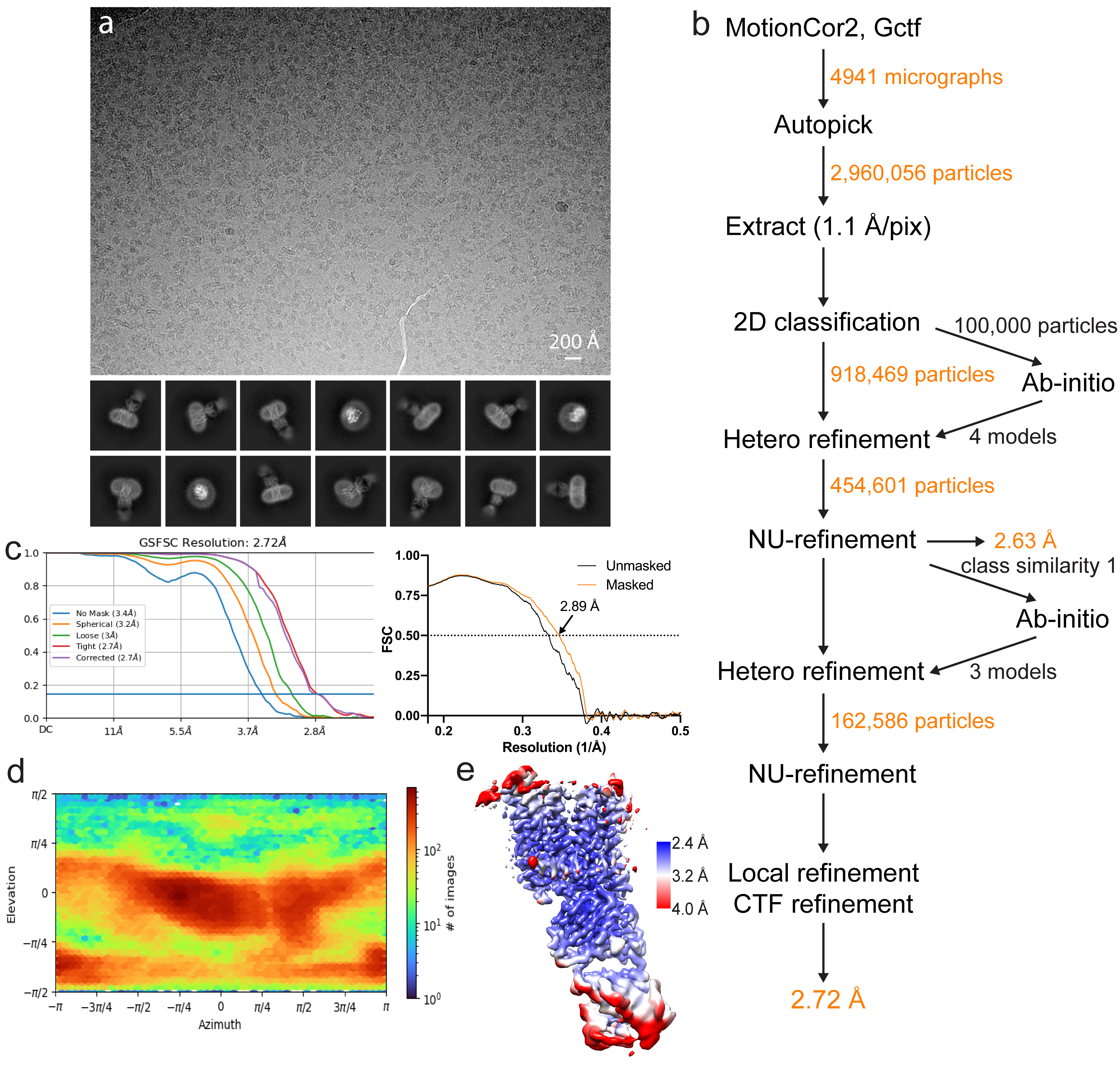

Supplement: S4 Fig — Related to Fig 3. (a) Representative electron micrograph (upper panel) and 2D class averages (lower panel). (b) Workflow of data processing for single-particle reconstruction. (c) The gold-standard Fourier shell correlation (FSC) curves for the final map (left panel) and map-to-model FSC curves (right panel). (d) Direction distribution of particles used in the final 3D reconstruction. (e) Local resolution map colored from 2.4 Å (blue) to >4.0 Å (red). Source data for (c) can be found in S1 Data. (TIF) [file pbio.3001936.s004.tif]

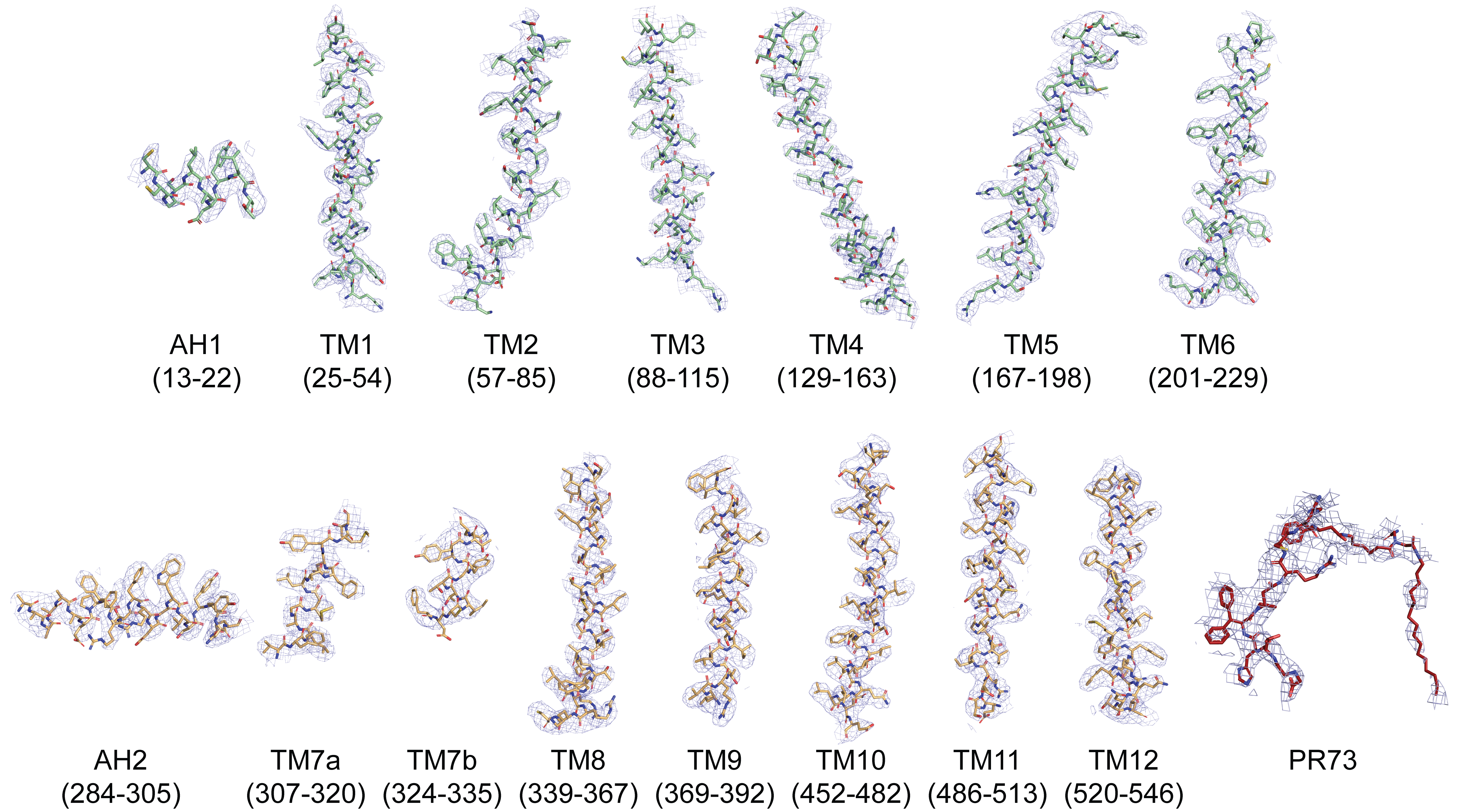

Supplement: S5 Fig — Related to Fig 3. Densities of HsFpn-PR73 are shown as blue mesh. Residues within the ranges indicated below are represented as sticks and colored in pale green, light orange, or brick red for the NTD, CTD, and PR73, respectively. (TIF) [file pbio.3001936.s005.tif]

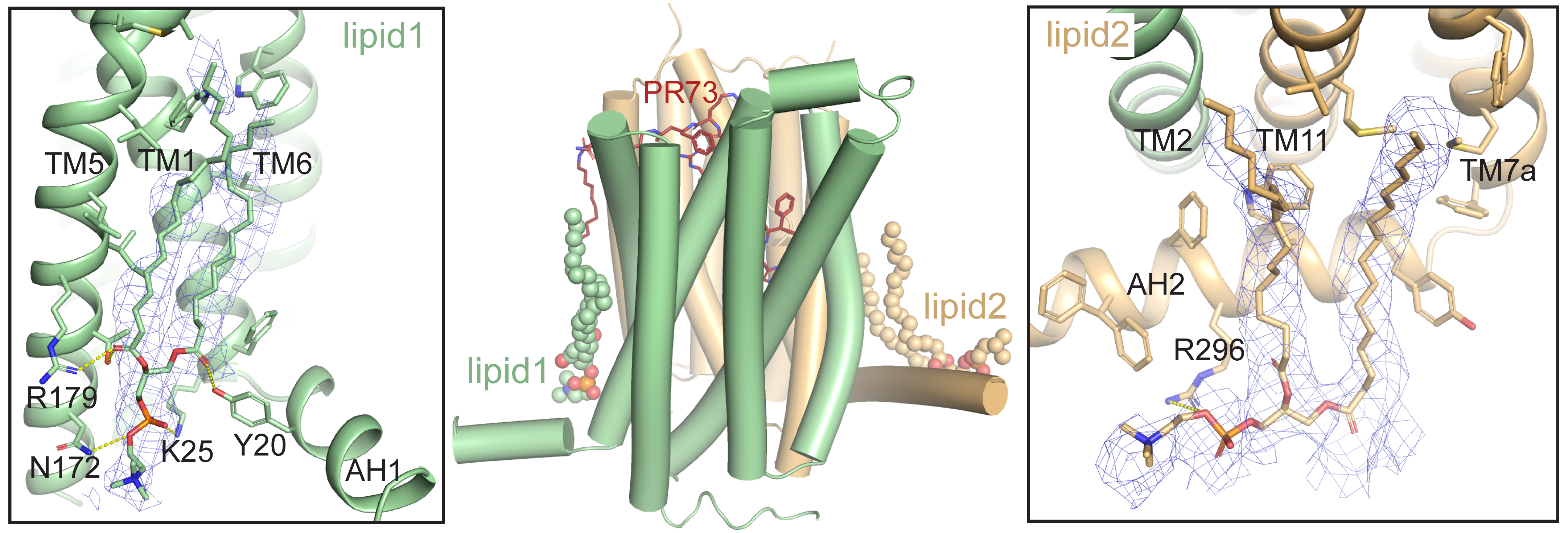

Supplement: S6 Fig — Related to Fig 3. Side view of 2 phospholipids (shown as spheres) located on the intracellular side in the HsFpn-PR73 structure (middle panel). Interactions of these lipids with residues of HsFpn (left and right panels). Side chains of residues within 4 Å from the lipids are shown as sticks. Hydrophilic interactions are indicated with yellow dashed lines. Densities of the lipids are contoured at 3.5σ as blue mesh. (TIF) [file pbio.3001936.s006.tif]

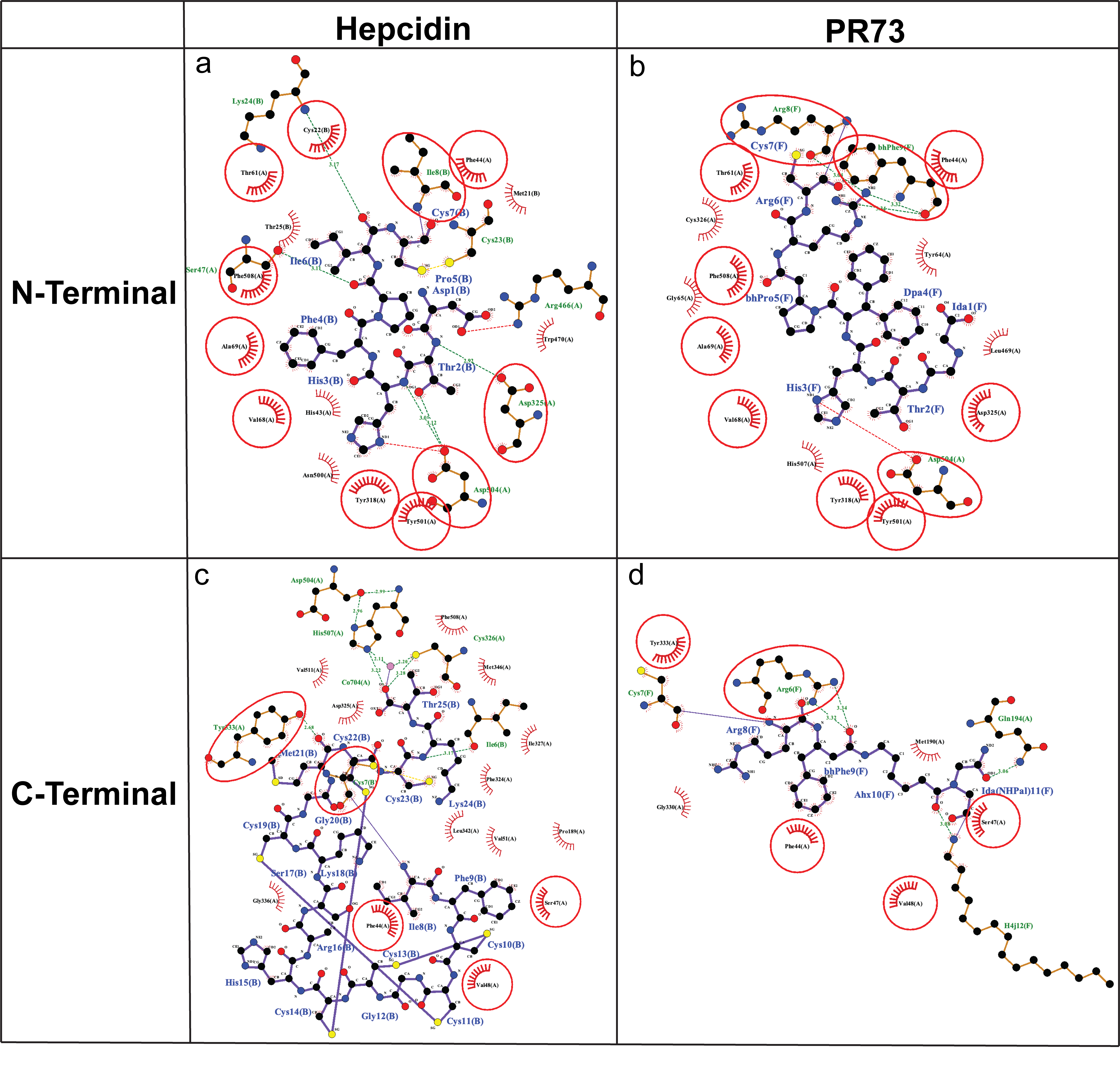

Supplement: S7 Fig — Ligplot of hepcidin (a and c) and PR73 (b and d) showing interactions in the binding pocket of HsFpn. The peptide is shown as purple sticks. Interactions common in both structures are marked by red circles. (TIF) [file pbio.3001936.s007.tif]

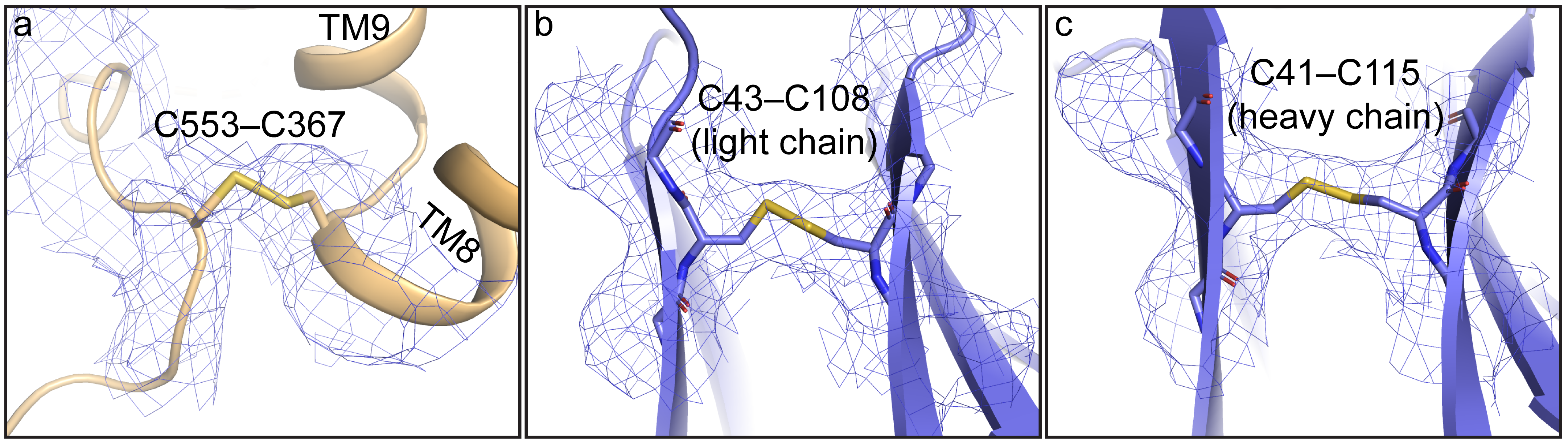

Supplement: S8 Fig — Related to Fig 5. (a) Cys553 near the C-terminus of HsFpn forms a disulfide bond with Cys367 between TM8 and TM9. Disulfide bonds in the light chain (b) and heavy chain (c) of the Fab. All density maps are contoured at 4.5σ as blue mesh. (TIF) [file pbio.3001936.s008.tif]

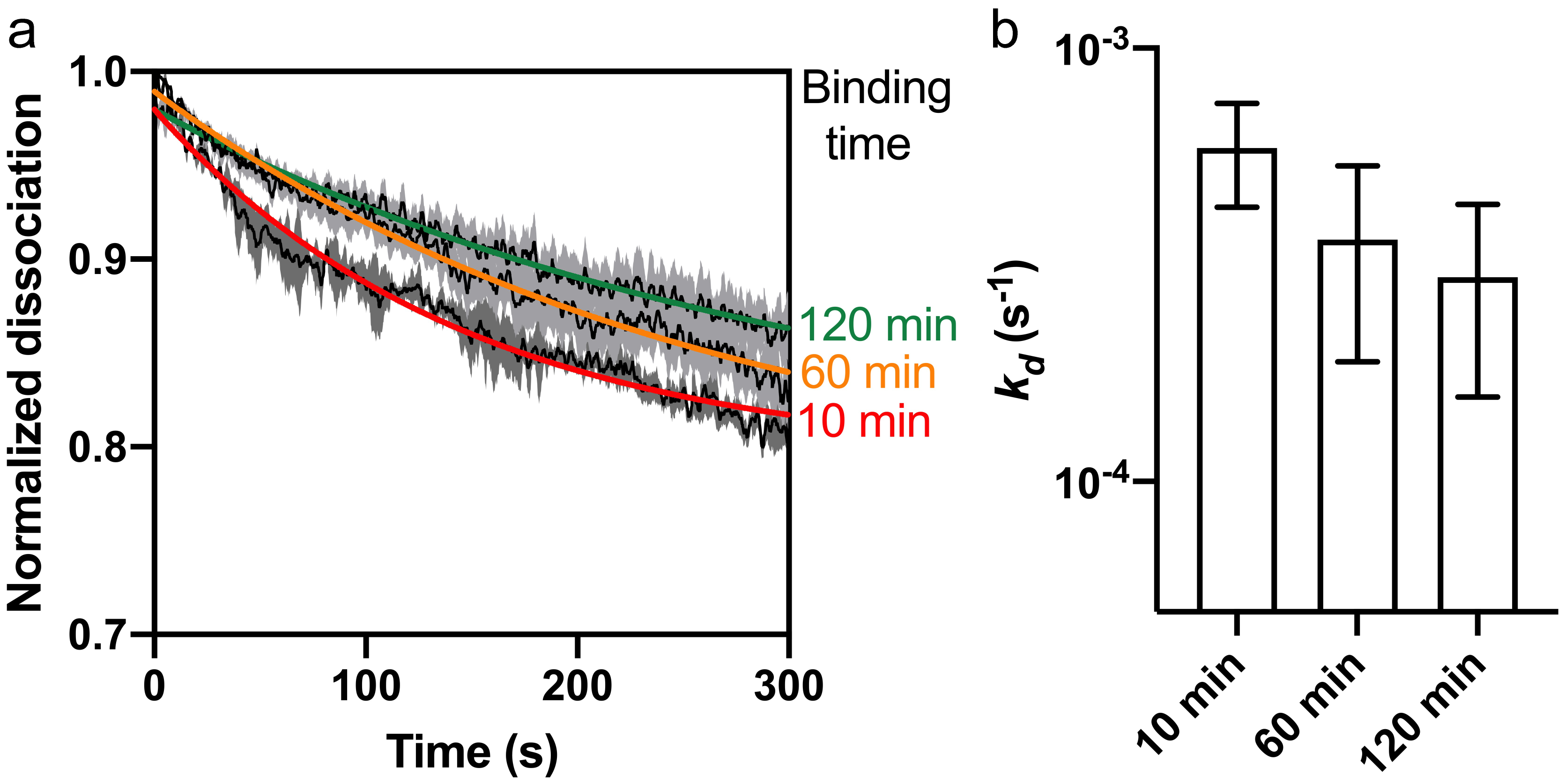

Supplement: S9 Fig — Related to Fig 6. (a) Dissociation phase of BLI assay is shown as mean (solid black line) and SD (gray shadow) at each point. The fit to data is shown as a colored curve of red, yellow, and green for incubation time of 10, 60, and 120 min, respectively. (b) Dissociation rate constants (kd) of HsFpn to PR73 with different lengths of incubation time. The height of the bar graphs represents the mean of at least 3 measurements and the error bar SEM. Source data for (b) can be found in S1 Data. (TIF) [file pbio.3001936.s009.tif]

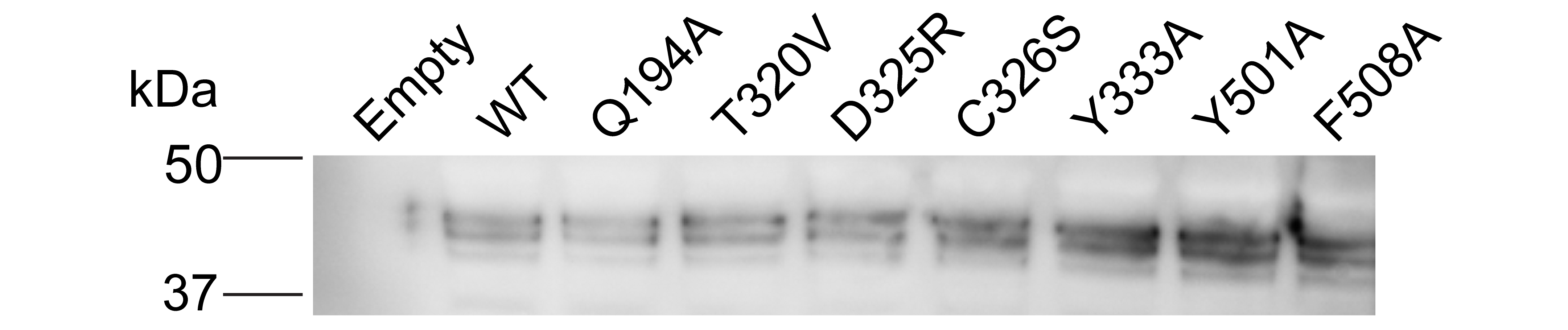

Supplement: S10 Fig — Related to Fig 7. (TIF) [file pbio.3001936.s010.tif]

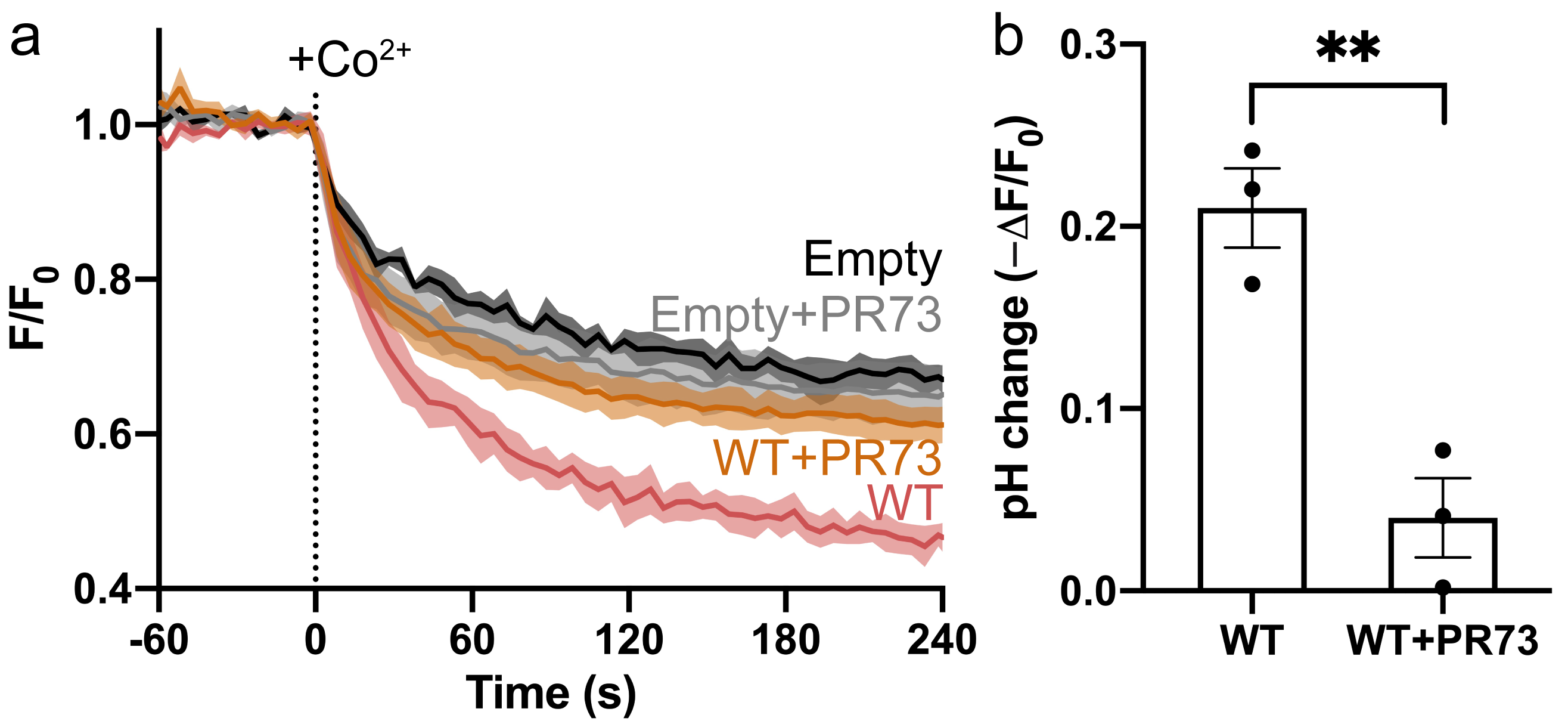

Supplement: S11 Fig — Related to Fig 7. (a) Co2+ import-induced H+ export by Fpn in HEK cells indicated by fluorescence change (F/F0) of a pH-sensitive dye (pHrodo Red) loaded inside cells. A total of 500 μM of Co2+ was administered at time zero. The solid lines represent the mean of 4 repeats and the shaded areas the SD. (b) Fpn-specific pH changes from data in (a). Each bar represents the change in fluorescence after subtraction of the fluorescence change in empty vector control cells. Data is plotted as means (n = 3) with error bars representing the SEM. Unpaired Student’s t test, p = 0.0052. Source data for (a–b) can be found in S1 Data. (TIF) [file pbio.3001936.s011.tif]

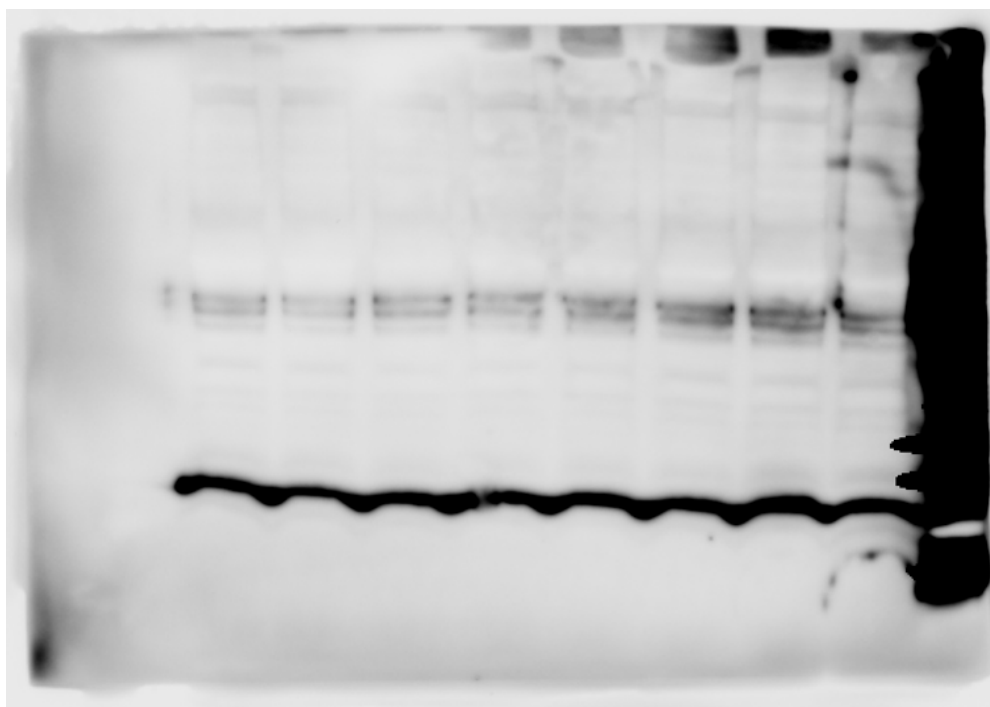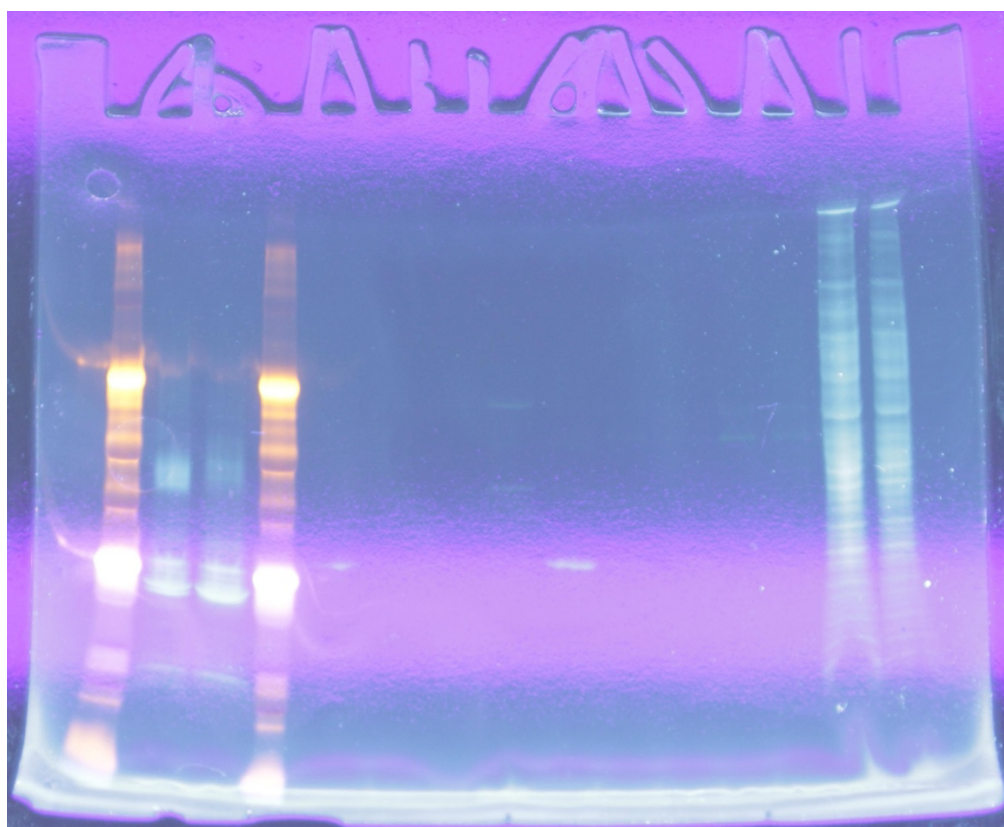

Supplement: S1 Raw image — (PDF) [file pbio.3001936.s015.pdf]
