# Supplementary material for: Structural basis of ferroportin inhibition by minihepcidin PR73
Source: PLoS Biol. 2023 Jan 17;21(1):e3001936. doi: 10.1371/journal.pbio.3001936 (PMC9882908; doi:10.1371/journal.pbio.3001936)
Supplement: S1 Table — (DOCX) [file pbio.3001936.s012.docx]

**S1 Table. Summary of cryo-EM data collection, processing, and refinement.**

| **Sample** | **HsFpn-Co^2+^-11F9** | **HsFpn-PR73-11F9** |
| --- | --- | --- |
| **Cryo-EM Data Collection** |  |  |
| Voltage (kV) | 300 | 300 |
| Magnification (x) | 81,000 | 81,000 |
| Pixel Size (Å) | 1.08 | 1.10 |
| Electron exposure (e^-^/Å^2^/frame) | 1.25 | 1.25 |
| Defocus range (µm) | [-2.25, -1.0] | [-2.0, -0.8] |
| Number of image stacks | 4,251 | 4,941 |
| Number of frames per stack | 40 | 40 |
| **Cryo-EM Data Processing** |  |  |
| Initial number of particles | 2,175,353 | 2,960,056 |
| Final number of particles | 215,164 | 162,586 |
| Symmetry imposed | C1 | C1 |
| Map resolution (Å) | 3.0 | 2.7 |
| Map resolution range (Å) | 2.5 – 3.7 | 2.3 – 3.8 |
| FSC threshold | 0.143 | 0.143 |
| **Model Refinement** |  |  |
| Number of amino acids | 877 | 905 |
| Total non-hydrogen atoms | 6,185 | 6,569 |
| Average B factor (Å^2^) | 145.5 | 120.9 |
| Bond length RMSD (Å) | 0.003 | 0.003 |
| Bond angle RMSD (°) | 0.534 | 0.606 |
| Ramachandran Plot |  |  |
| Favored (%) | 97.4 | 96.6 |
| Allowed (%) | 2.6 | 3.4 |
| Outliers (%) | 0.00 | 0.00 |
| Rotamer outliers (%) | 0.46 | 0.33 |
| Clash score | 5.65 | 4.31 |
| MolProbity Score | 1.44 | 1.31 |
