# Supplementary material for: Structural basis of ferroportin inhibition by minihepcidin PR73
Source: PLoS Biol. 2023 Jan 17;21(1):e3001936. doi: 10.1371/journal.pbio.3001936 (PMC9882908; doi:10.1371/journal.pbio.3001936)
Supplement: S2 Table — Related to Fig 6. (DOCX) [file pbio.3001936.s013.docx]

**S2 Table. Binding affinities of PR73 to Fpn measured by Octet BLI. Related to Fig 6.**

| **Interaction type** | **Sample name** | ***K_D_* (M)** |
| --- | --- | --- |
| Disulfide bridge by C326 | WT | 3.70 ± 0.66 × 10^-8^ |
|  | WT + BME | 1.39 ± 0.51 × 10^-6^ |
|  | WT + Co^2+^ | 8.83 ± 1.12 × 10^-7^ |
|  | C326S | 2.39 ± 1.64 × 10^-6^ |
| Hydrophilic interactions | Q194A | 6.11 ± 3.24 × 10^-6^ |
|  | T320V | 2.40 ± 0.80 × 10^-7^ |
|  | D325A | 4.18 ± 2.65 × 10^-7^ |
| Hydrophobic interactions | Y64N | 4.59 ± 1.60 × 10^-7^ |
|  | Y333A | 1.40 ± 0.23 × 10^-7^ |
|  | Y501A | 7.49 ± 0.98 × 10^-7^ |
|  | F508A | 2.01 ± 0.50 × 10^-7^ |
